# Tumor slice cultures enable rapid ex vivo profiling of patient-specific drug sensitivity in pancreatic ductal adenocarcinoma

**DOI:** 10.1038/s41698-026-01663-z

**Published:** 2026-08-20

**Authors:** Benjamin Heckelmann, Daniel Fetzner, Olga Lapshyna, Ashinikumar Singh Hidam, Annika Brauer, Anna Maxi Wandmacher, Benedikt Färber, Jannis Duhn, Meike ten Winkel, Louisa Bolm, Kim Honselmann, Steffen Deichmann, Christoph Röcken, Darko Carstven, Jens Marquardt, Corinna Keber, Timo Gemoll, Susanne Sebens, Tobias Keck, Ulrich Wellner, Rüdiger Braun

**Affiliations:** 1 Department of Surgery, University of Luebeck, Luebeck, Germany; 2 Institute for Experimental Tumor Research, Kiel University, University Hospital Schleswig-Holstein, Kiel, Germany; 3 Department of Internal Medicine II, Division of Hematology and Oncology, University Hospital Schleswig-Holstein, Kiel, Germany; 4 Department of Hematology and Oncology, University of Luebeck, Luebeck, Germany; 5 Institute of Pathology, Kiel University, University Hospital Schleswig-Holstein, Kiel, Germany; 6 Department of Internal Medicine I, University of Luebeck, Luebeck, Germany; 7 Institute of Pathology, University Hospital of Giessen-Marburg, Marburg, Germany

**Keywords:** Cancer, Medical research, Oncology

## Abstract

Pancreatic ductal adenocarcinoma (PDAC) is characterized by chemotherapy resistance, partly driven by its dense and heterogeneous tumor microenvironment (TME). Since most preclinical PDAC models inadequately capture the tissue architecture, their translational value for therapeutic testing remains limited. This study investigated organotypic tissue slice cultures (OTSCs), which preserve the multicellular tissue architecture, as a rapid platform for personalized ex vivo drug response profiling. OTSCs were generated from 27 resected PDAC specimens. Following workflow establishment and quality control, ex vivo drug profiling was performed in 15 patients using gemcitabine, gemcitabine plus paclitaxel, and FOLFIRINOX. Treatment response was assessed by quantitative digital pathology, and an ex vivo sensitivity score (EVSS) was defined. Clinical correlations with longitudinal follow-up were assessed in ten patients. OTSCs preserved tissue architecture and revealed interpatient heterogeneity. In exploratory analyses of clinically matched patients, ex vivo sensitivity was associated with prolonged PFS (median 445 vs. 141 days, log-rank *p* = 0.0027; HR per 10 EVSS points 0.625; 95% CI 0.484–0.807, *p* = 0.00032), while lower preoperative CA 19-9 levels and higher GATA6 expression were associated with higher EVSS and longer overall survival. OTSC-based profiling enables rapid, patient-specific drug response assessment in PDAC and supports evaluation as a functional stratification tool.

## Introduction

Pancreatic ductal adenocarcinoma (PDAC) is projected to become the second leading cause of cancer-related deaths in 2030, with a 5-year overall survival (OS) of approximately 10%^1,2^. In addition to the often-late diagnosis, which leads to curative surgical resection in only 20% of cases^2^, frequent metastasis, marked tumor heterogeneity, a dense desmoplastic reaction, and an immunosuppressive tumor microenvironment (TME) contribute to chemotherapy resistance^3^. The polychemotherapy regimen (modified) FOLFIRINOX, consisting of folinic acid/5-fluorouracil/irinotecan/oxaliplatin (FFX), gemcitabine monotherapy (GEM) or GEM combinations, such as GEM with albumin-bound paclitaxel (GnP) and GEM with capecitabine (GEM/CAP) are recommended as first-line therapies in American and German guidelines^4,5^. The choice of treatment depends on the performance status of the patient and the stage of the disease. The current standard of care is based on a limited number of therapeutic agents and combinations^6^.

Over the past decade, substantial effort has been invested in gaining a more profound comprehension of the molecular biology of individual tumors. To improve the dismal prognosis of patients diagnosed with PDAC, several studies have explored the potential of a targeted, personalized approach over the traditional ‘one-size-fits-all’ strategies^7–9^. However, to date, only a few targeted molecular and immunotherapeutic options have succeeded in clinical trials and are FDA approved^6,10^. Those approved are only available for small subgroups of PDAC patients with a prevalence of approximately one percent^6^. In the largest targeted therapy program, the “Know your tumor registry”, only 282 (15.2%) of the 1856 included PDAC patients had actionable mutations. Only 46 patients (2.5%) received matched therapies^9^. Consequently, in addition to focusing on targeted approaches, current research increasingly aims to individualize classical (poly)chemotherapies. Ongoing studies, such as the COMPASS and ESPAC-6 trial^11,12^, attempt to stratify patient treatment based on transcriptomic PDAC subtypes^6^. Functional ex vivo tumor models may represent a complementary approach to explore patient-specific drug responses in a clinically relevant time frame.

It has been shown recently that the individual TME, immunity, tumor differentiation, and treatment response are linked to molecular PDAC subtypes^13–16^. Therefore, it appears essential to preserve diverse tumor ecosystems, encompassing cancer cells, cancer-associated fibroblasts (CAFs), pancreatic stellate cells, blood, immune cell populations, and extracellular matrix (ECM) components for translational response modeling. To overcome the limitations of previously established patient-derived culture systems, such as patient-derived xenografts (PDXs) and patient-derived organoids (PDOs), we and others described a method for generating PDAC organotypic tissue slice cultures (OTSCs)^17–20^. PDXs and PDOs mostly lack the human immunologically infiltrated stromal compartment^21,22^. In contrast, OTSCs preserve the entire original tissue architecture^17–20^. This enables short-term functional assessment of treatment effects within an intact TME.

This study aimed to explore the feasibility and translational relevance of OTSCs for ex vivo treatment response assessment in patients with surgically resected PDAC. We developed a time efficient ex vivo drug response platform using OTSCs, immunohistochemistry (IHC) and subsequent machine-learning-based digital image analysis (DIA)^23^ to characterize treatment-associated tissue responses. Using this OTSC drug testing platform we assessed the therapeutic impact of current standard treatment regimens, i.e., GEM, GnP, FFX. Correlations between ex vivo drug responses and clinical and histopathological response markers, as well as survival, were explored. Conclusively, this work provides proof-of-concept evidence for OTSC-based functional profiling as a potential tool for rapid and individualized assessment of ex vivo drug response in PDAC.

## Results

### Patient characteristics and OTSCs establishment

**Fig. 1:**
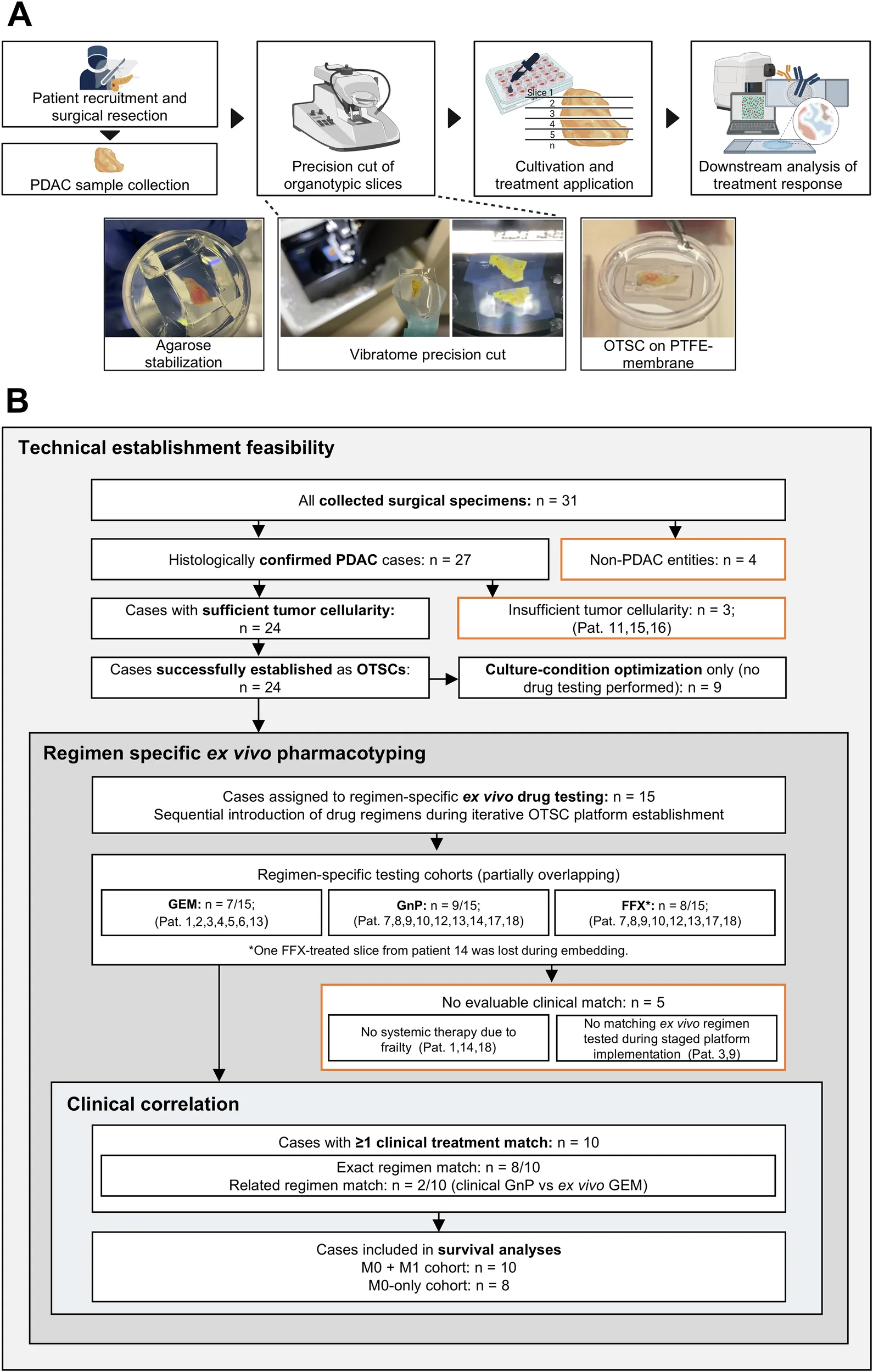
Workflow of PDAC OTSC ex vivo drug testing platform. **A** Generation of organotypic tissue slice cultures (OTSCs) from resected PDAC specimens. Tumor tissue was embedded in agarose, sectioned using a vibratome, and cultivated at the air-liquid interface on PTFE membranes followed by ex vivo drug treatment and histological analysis. Representative photographs illustrate agarose embedding, vibratome slicing, and OTSC cultivation. **B** CONSORT flow diagram of sample inclusion, feasibility assessment, and analysis sets. Drug testing was initiated after initial culture-condition optimization. Assignment to ex vivo drug testing reflected chronological case accrual rather than clinical or pathological selection.

**Fig. 2:**
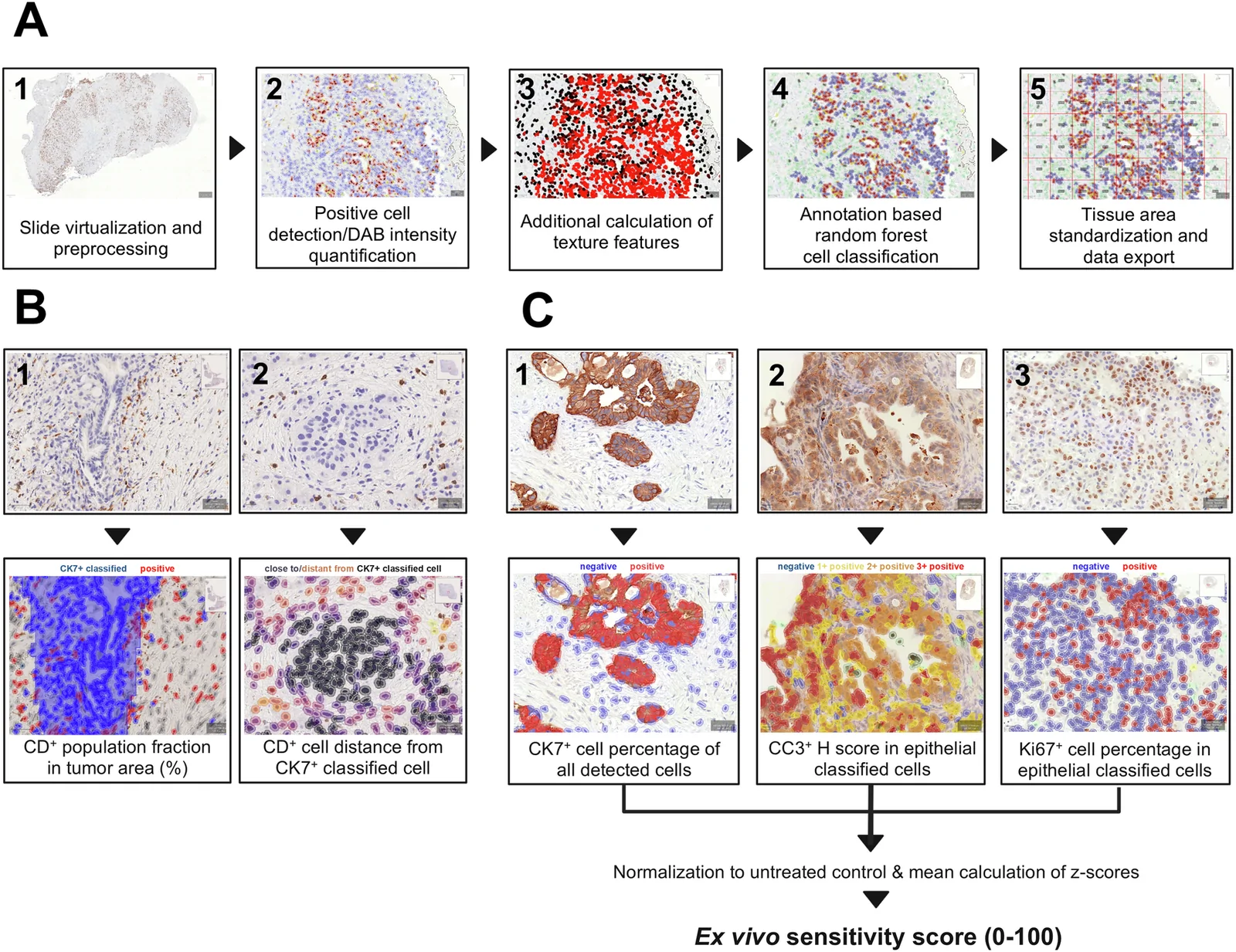
Digital pathology workflow in QuPath. **A** Slides were virtualized, stain vectors estimated, and tissues detected (1). Positive cells were identified by DAB intensity (2), further classified by morphological and texture features (3), and assigned to compartments by a random forest model (4). All readouts were normalized to tissue area before data export (5). **B** Quantitative mapping of immune cells relative to tumor. Distances between DAB-positive immune cells and epithelial (CK7 classified) tumor cells were visualized as gradients (black = proximal, yellow = distal) (1). Infiltration of CD positive immune cells into tumor areas was quantified as percentage (2). **C** Quantification of CK7-positive tumor fraction (red = positive, blue = negative), CC3 H-score (blue = negative, yellow = 1+, orange = 2+, red = 3+; 2), and Ki67-positive tumor cells (red = positive, blue = negative; 3). Values were normalized to untreated controls, z-standardized, and integrated into an equally weighted, rank-based Ex Vivo Sensitivity Score (EVSS, 0–100).

During the study period, 31 pancreatic tissue specimens were processed for OTSC generation (Fig. 1A, B). After final histopathological evaluation, 27 specimens were confirmed as PDAC, of which 24 showed sufficient tumor cellularity and were successfully established as OTSCs. Nine PDAC specimens were used during an initial run-in phase for optimization and quality control of tissue processing, cultivation conditions, and digital pathology-based readout generation without regimen-specific drug testing (Fig. 2), whereas 15 PDAC specimens underwent regimen-specific ex vivo drug-treatment experiments. Due to the sequential introduction of chemotherapy regimens during iterative platform establishment, treatment-testing cohorts partially overlapped. Clinical treatment matching was achievable in 10 patients, including 8 exact regimen matches and 2 related gemcitabine-based matches. Clinicopathological characteristics and baseline statistics: Table 1 and Supplementary Table S1; translational workflow, feasibility assessment, and analysis sets: Fig. 1B.

**Table 1:** Clinicopathological characteristics and baseline comparisons

Characteristic	Cultivation establishment (PDAC, *n* = 27)	Pharmacotyping cohort (evaluable, *n* = 15)	*p*-value^a^	Clinical correlation cohort (*n* = 10)	*p*-value^b^
Age, years			0.788		0.124
Median	72	72		66	
Range	52–86	55–86		55–86	
Sex, *n* (%)			1.000		1.000
Female	11 (41)	6 (40)		4 (40)	
Male	16 (59)	9 (60)		6 (60)	
UICC stage, *n* (%)			1.000		1.000
I–II	15 (56)	8 (53)		5 (50)	
III	9 (33)	5 (33)		3 (30)	
IV	3 (11)	2 (13)		2 (20)	
M status, *n* (%)			1.000		0.524
M0	24 (89)	13 (87)		8 (80)	
M1	3 (11)	2 (13)		2 (20)	
R status, *n* (%)			1.000		0.56
R0	16 (59)	9 (60)		6 (60)	
R1	7 (26)	4 (27)		2 (20)	
Not assessable (metastatic sample)	3 (11)	2 (13)		2 (20)	
Missing	1 (4)	0 (0)		0 (0)	
Grade, *n* (%)			0.107		0.282
G1	0 (0)	0 (0)		0 (0)	
G2	12 (44)	4 (27)		3 (30)	
G3	10 (37)	8 (53)		4 (40)	
Not assessable (metastatic sample)	3 (11)	2 (13)		2 (20)	
Not assessable (post-treatment)	2 (7)	1 (7)		1 (10)	
Pre-op. CA 19-9, U/ml			0.578		0.179
Median (IQR)	Not reported	484.5		379.5	
Range	Not reported	20.6–10,000		20.6–3474	
Available, *n* (%)	16 (59)	14 (93)		10 (100)	

Tumor stage according to the 8th AJCC/UICC classification^57^. All pathological parameters were retrospectively extracted from routine pathology reports. Tumor grading was not applicable for metastatic samples and post-treatment specimens due to limited interpretability of conventional histopathological grading following therapy-associated tissue alterations. Pre-operative CA 19-9 levels were not systematically assessed during the initial platform establishment phase and are therefore reported descriptively only for the pharmacotyping and clinical correlation cohorts. P-values are exploratory and were calculated using Fisher’s exact tests for categorical variables and Wilcoxon rank-sum tests for continuous variables.

^a^Exploratory comparison between the pharmacotyping cohort (*n* = 15) and excluded PDAC cases without evaluable pharmacotyping (*n* = 12).

^b^Exploratory comparison between the clinical correlation cohort (*n* = 10) and unmatched pharmacotyped cases (*n* = 5).

### Tumor viability is sustained during cultivation

**Fig. 3:**
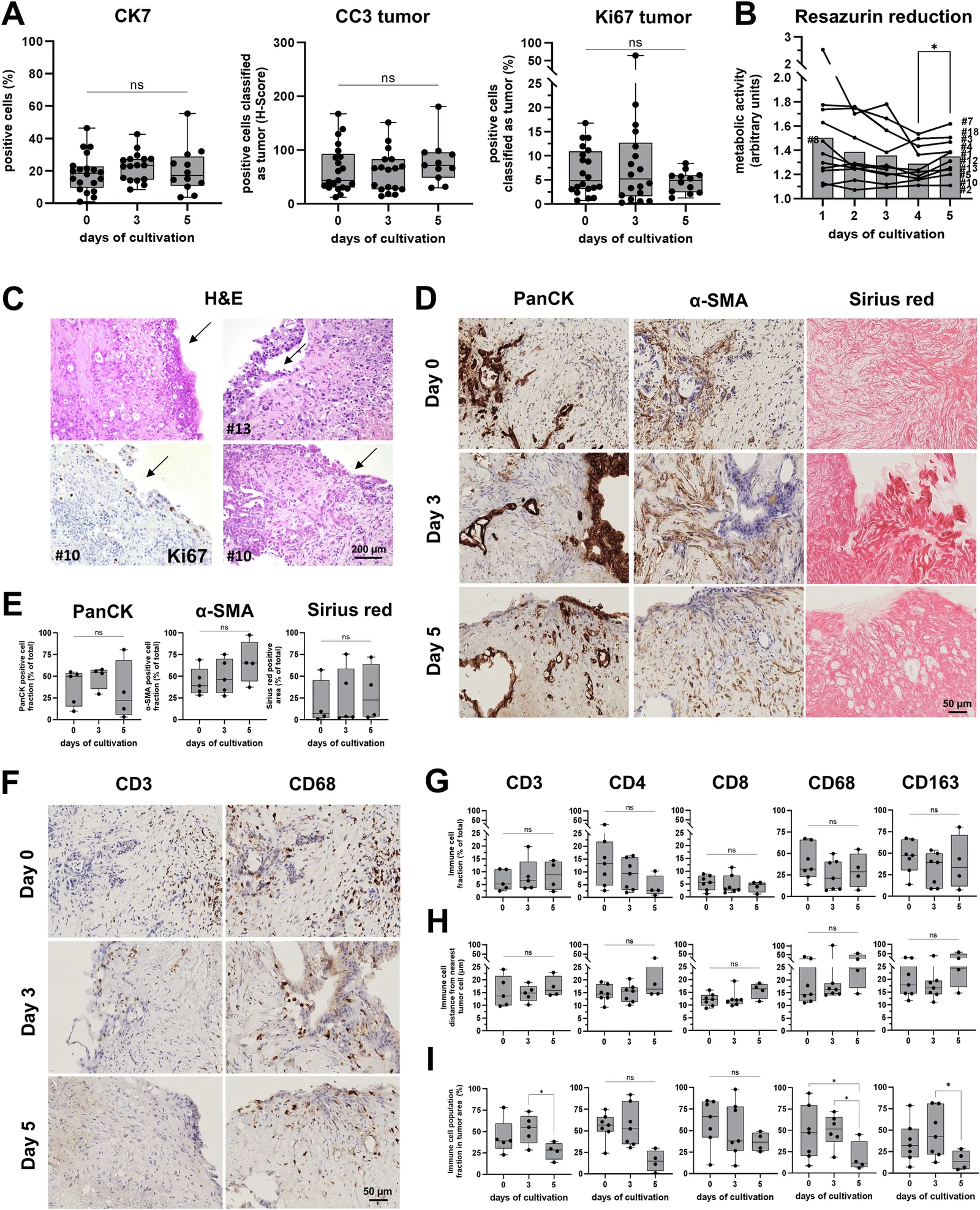
Cultivation effect and stability of the tumor microenvironment (TME). **A** CK7-positive tumor fraction (%), CC3 H-score, and Ki67-positive tumor cells (%) at days 0, 3, and 5 of cultivation (*n* = 22). **B** Viability assay based on resazurin metabolism in OTSC media (*n* = 11), expressed as fluorescence ratio to blank controls. **C** Representative examples of proliferative surface outgrowth zones observed during OTSC cultivation. H&E and corresponding Ki67 staining illustrate viable proliferative tumor-cell expansion at the tissue surface facing the culture medium (arrows). Scale bar, 200 µm. **D**, **E** Representative IHC for PanCK, α-SMA, and Sirius red at days 0, 3, and 5, with quantification in *n* = 5 patients (% positive cells or area). Scale bar, 50 µm. **F** Representative IHC images of CD3 positive T cells and CD68 positive macrophages at days 0, 3, and 5. Scale bar: 50 µm. **G**–**I** Quantification of immune cell fractions, distances to nearest tumor cell, and infiltration into CK7 positive regions (*n* = 7). Mixed-effects ANOVA with Tukey correction.

The median yield per specimen was eleven OTSCs (range: 6–54). The median area of Hematoxylin-eosin-stained sections was 3.44 mm^2^ (range: 0.12–79.21 mm^2^). The overall tissue structure remained unchanged over the five-day cultivation period as assessed by H&E staining. No significant changes in cytokeratin 7 (CK7), Ki67, or cleaved caspase 3 (CC3) expression were observed (*n* = 22), consistent with results from the resazurin viability assay (Fig. 3A, B). Of note, new Ki67-positive cancer cell populations on the OTSC surface emerged on days 3 and 5 (exemplary images shown in Fig. 3C). This phenomenon was previously described as “cancerous outgrowth”, indicating active proliferation^19,24^.

### Structural preservation of multicellular TME architecture

To evaluate the structural preservation of distinct components of the multicellular TME, markers for epithelial (tumor) cells (pan-cytokeratin) and stromal markers (α-SMA, Sirius Red) were quantified and revealed no significant alterations (Fig. 3D, E). Tumor immune infiltrates (CD3, CD4, CD8, CD68, CD163) were consistently detectable (Fig. 3F–I), with macrophages (CD68 and M2-marker CD163) exceeding T cell quantity (CD3, CD4 and CD8). Exemplarily, CD68 positive macrophages decreased from 31.74% on day 0 to 21.17% on day 3, before rising to 28.67% on day 5, while the percentage of CD3 positive T cells increased from 5.18% to 8.79% over the same period (Fig. 3G). The median spatial distance between the immune cell populations analyzed and the nearest tumor cell remained consistent throughout the total cultivation period (Fig. 3H). Infiltration analysis showed a trend for peripheral relocation by day five (Fig. 3I). However, the consistent ratio of extra- to intratumoral localization between days 0 and 5 suggest the relative preservation of the spatial immune-tumor architecture for a minimum of three days.

### OTSCs respond specifically ex vivo to distinct drugs

**Fig. 4:**
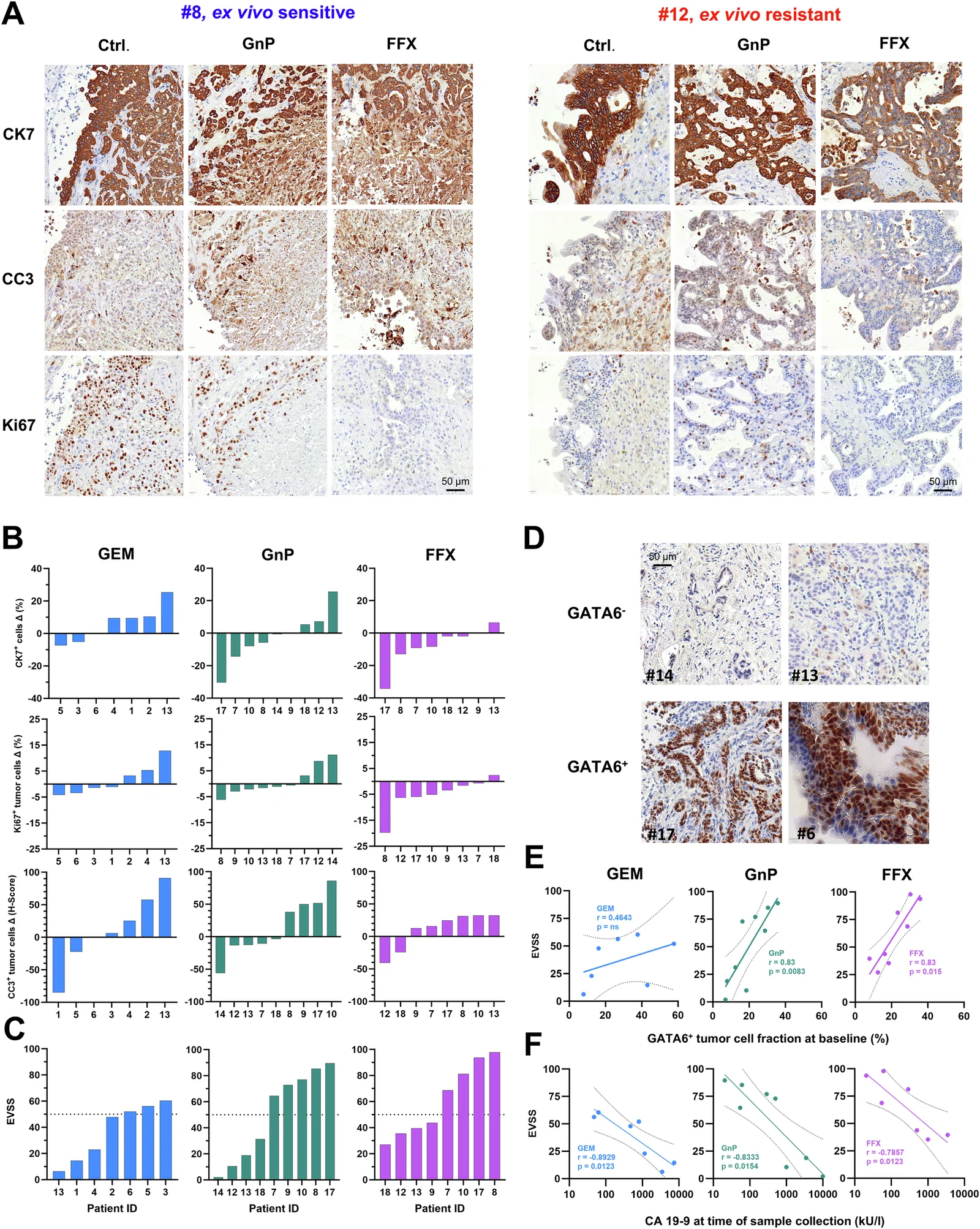
Ex vivo drug response and biomarker correlations in PDAC OTSCs. **A** Representative IHC for CK7, CC3, and Ki67 after 48 h exposure with untreated control, gemcitabine + albumin-bound paclitaxel (GnP), and FOLFIRINOX (FFX). Scale bar, 50 µm. **B** Quantification of CK7 (% of cells), CC3 (H-score of tumor cells), and Ki67 (% of tumor cells) across patients (GEM: *n* = 7, GnP: *n* = 9, FFX: *n* = 8). Effects were calculated relative to local untreated controls to correct for cultivation effects. **C** Ex Vivo Sensitivity Scores (EVSS, 0–100) per patient and regimen. EVSS was calculated as an equally weighted, rank-based integration of CK7, CC3, and Ki67 changes after normalization to untreated controls. Ex vivo sensitivity cut-off was defined as median across treatment options at EVSS 50 (dotted line) **D** Representative IHC for baseline GATA6 expression at day 0. **E** Spearman correlations between baseline GATA6 tumor cell fraction and EVSS for GEM, GnP, and FFX. **F** Spearman correlations between preoperative CA19-9 levels at sample collection and EVSS for GEM, GnP, and FFX.

To evaluate the feasibility of OTSCs to capture inter-patient heterogeneity in treatment response ex vivo, we assessed drug-induced effects across multiple clinically used regimens. Accordingly, OTSCs from 15 patients were exposed ex vivo to clinically used chemotherapeutic regimens, including GEM, GnP, and FFX, using concentrations selected based on clinically relevant peak plasma levels (C_max_)^25^ and prior ex vivo studies^18,24,26^. Due to the exploratory nature of study design, but also variability in tissue yield and slice availability, not all chemotherapeutic regimens were tested for every patient. Ex vivo drug sensitivity was quantified based on CK7 positive cell fraction, CC3 H-Score, and Ki67 positive tumor cell fraction (Fig. 2C), exemplarily shown for patient #8 (ex vivo sensitive) and patient #12 (ex vivo resistant) (Fig. 4A). The expression scores were combined to the proposed ex vivo sensitivity score (EVSS; range 0 (worst response) to 100 (best response)) (Fig. 4B, C). We observed striking heterogeneity of the EVSS across patients and treatment regimens.

FFX induced the most pronounced effects, including decreased CK7 positive fraction (median −5.16%; Fig. 4B) and increased CC3 H-Score compared to GEM and GnP. However, in some cases, GEM and GnP induced stronger apoptosis. Proliferation was most effectively decreased by FFX (median −4.28%) versus GEM and GnP (median both −1.05%) (Fig. 4B). Marked inter-patient variability in EVSS values was observed across all regimens, including distinct response patterns in the two metastatic OTSCs (#12 and #13), which exhibited pronounced proliferation inhibition under FFX compared with GnP. Overall, OTSCs showed on average the highest median EVSS values under FFX (mean EVSS: 60.93) compared with GnP (50.23) and GEM (37.20), although regimen-level comparisons were exploratory and descriptive only (Fig. 4C; for additional information, see Supplementary Table S2).

### EVSS correlates with the expression of classical-like subtype marker GATA6

Distinct molecular PDAC subtypes are associated with differences in drug response and prognosis^6,27^. The tumor-associated expression of the differentiation marker GATA6 can distinguish between classical-like and basal-like subtypes in advanced PDAC^28^. In our cohort, GATA6 IHC positivity ranged between 6.95% (patient #14) and 57.67% (patient #6) (Fig. 3D). Consistent with its known prognostic role, higher tumor cell GATA6 expression was associated with longer OS in our cohort (univariable Cox analysis; HR per 10% increase = 0.28, 95% CI 0.11–0.74, *p* = 0.01). Baseline GATA6 expression was positively correlated with the EVSS in GnP- and FFX-treated OTSCs (FFX: r = 0.83, *p* = 0.015; GnP: r = 0.83, *p* = 0.0083), with a similar but non-significant trend observed for GEM (Fig. 4E). These findings indicate that the EVSS may capture tumor-intrinsic lineage biology reflected by GATA6 expression and support the biological plausibility of the EVSS.

### Correlation of EVSS with CA 19-9 levels

Pre-operative carbohydrate antigen 19-9 (CA19-9) levels were inversely associated with EVSS across treatment regimens, with higher serum levels corresponding to lower ex vivo drug sensitivity (Fig. 4F and Supplementary Fig. S1). This relationship was consistent in regimen-specific analyses (GEM: r = −0.89, *p* = 0.012, *n* = 7; GnP: r = −0.83, *p* = 0.015, *n* = 8; FFX: r = −0.79, *p* = 0.048, *n* = 7). Linear regression using log-transformed CA19-9 values demonstrated consistent negative slopes and good model fit (R² = 0.72–0.77 across regimens), confirming robustness against the distribution of CA19-9. Exploratory correlative analyses further demonstrated inverse associations between pre-operative CA19-9 levels and GATA6 expression in the GnP and FFX cohorts, whereas no clear association was observed in the GEM cohort, suggesting partially overlapping but regimen-dependent biological relationships between these exploratory biomarkers (Supplementary Fig. S1). Higher pre-operative CA19-9 levels were also associated with worse OS (HR per 10-fold increase = 14.0, 95% CI 2.39–82.09, *p* = 0.003). Together, these exploratory findings might indicate that higher pre-operative tumor burden is linked to reduced ex vivo drug sensitivity and poorer survival. This supports the biological consistency between EVSS, GATA6 expression, and CA 19-9 levels.

### Correlation of EVSS with clinical response and survival parameters

**Fig. 5:**
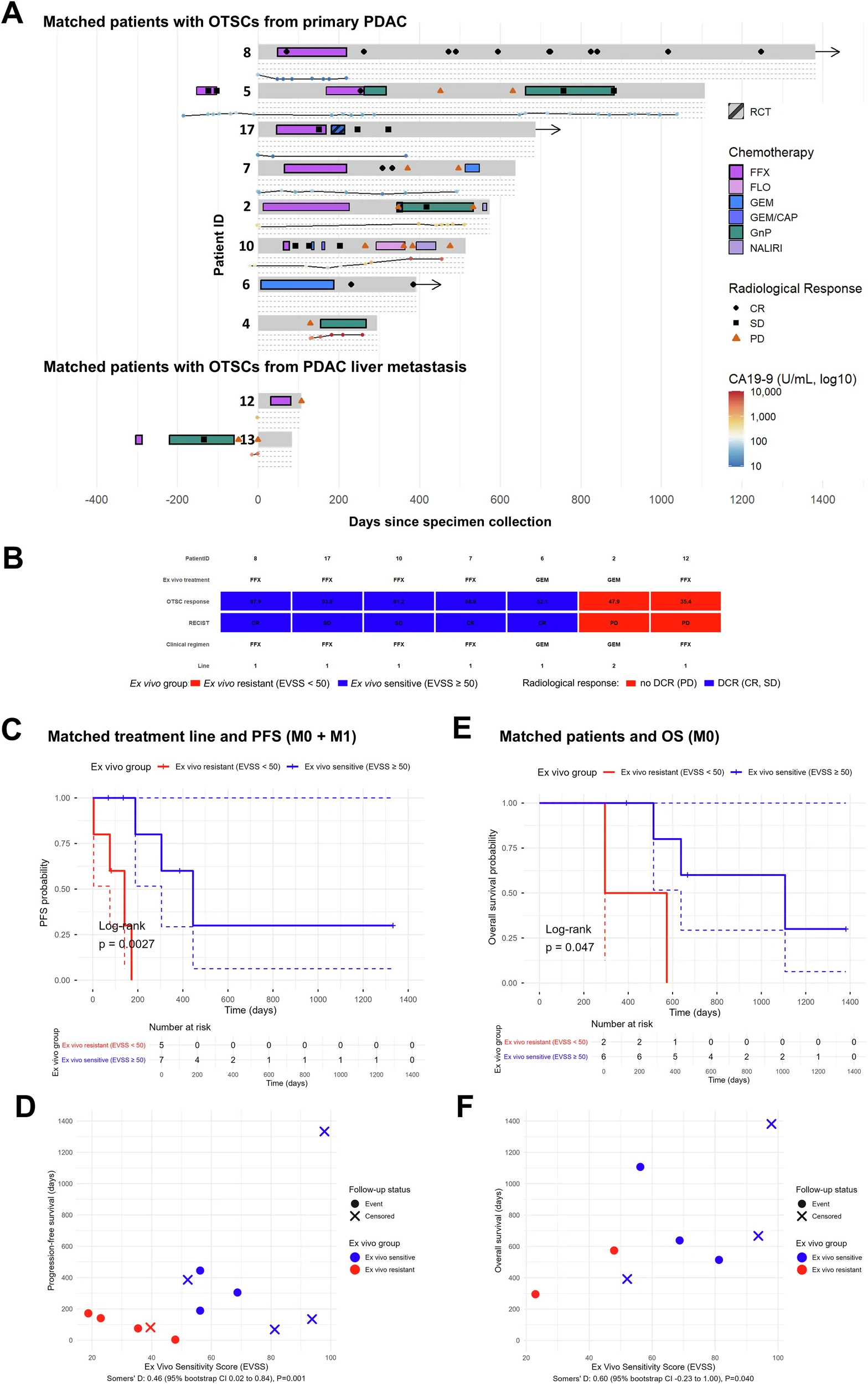
Ex vivo sensitivity correlates with treatment response and survival in matched patients. **A** Clinical courses of patients with OTSCs tested for treatment options matching at least one of the clinical treatment lines. Upper panel: primary PDAC (M0); lower panel: liver metastases (M1). Gray bars indicate overall survival (OS) after specimen collection (day 0). Colored bars represent administered chemotherapy (FFX, FLO, GEM, GEM/CAP, GnP, NALIRI). Radiological responses (CR complete response, SD stable disease, PD progressive disease) and log10-transformed CA19-9 levels are shown. Arrows indicate censored observations. RCT, radiochemotherapy. **B** Matched ex vivo and clinical treatments, including exact and approximate pharmacologic matches: patient ID, tested regimen ex vivo, OTSC response (EVSS, EVSS classification ≥50 (blue, sensitive) vs <50 (red, resistant)), Radiological RECIST status, DCR (CR, SD): blue; no DCR (PD): red), clinical treatment regimen, and treatment line. DCR, disease control. **C** Kaplan–Meier analysis of treatment-specific progression-free survival. (PFS; M0 + M1) stratified by ex vivo sensitivity (EVSS ≥ 50 vs <50) in *n* = 12 treatment lines of 10 patients. Log-rank test. **D** Correlation of EVSS with PFS shown as Somers’ D with bootstrap confidence intervals. **E** Kaplan–Meier analysis of OS (M0) stratified by ex vivo sensitivity in *n* = 8 patients. Log-rank test. **F** Correlation of EVSS with OS shown as Somers’ D with bootstrap confidence intervals.

To explore whether ex vivo drug-response patterns derived from OTSCs are associated with clinical outcomes, we performed correlation analyses between EVSS and distinct survival parameters (Fig. 5, Supplementary Fig. S2) focusing on treatment-line-specific PFS. Consistent with the heterogeneity observed in the ex vivo assays, clinical courses and survival outcomes varied substantially between patients (Fig. 5A & Supplementary Fig. S3). OTSCs were classified into ex vivo sensitive and resistant groups using the predefined EVSS median cutoff of 50. Patients who received systemic therapy corresponding to the ex vivo setting were identified (*n* = 10 patients contributing 12 evaluable treatment-line observations). A comprehensive patient-level and treatment-line-level overview of all pharmacotyped cases, comprising included and non-included clinical correlation cases, exact and approximate regimen matching, and timing of tissue acquisition relative to systemic therapy, is provided in Supplementary Tables S2, S3. Due to the iterative implementation of the OTSC pharmacotyping workflow, ex vivo treatment-testing cohorts partially overlapped and not all regimens were assessed in all patients. Drug regimens were introduced sequentially over time, with gemcitabine monotherapy tested in early cases before implementation of GnP- and FFX-based pharmacotyping. For gemcitabine-based regimens, approximate pharmacologic matching was accepted when GEM monotherapy had been tested ex vivo but patients received gemcitabine-based combination therapy clinically (*n* = 3 matched lines from 2 patients; #4 and #5).

In the disease control rate (DCR) concordance analysis of first restaging after chemotherapy initiation, cases without formal restaging imaging (patient #4, #5) and post-treatment tissue acquisition (patient #13) were excluded. Among the remaining fully evaluable treatment-naive cases, treatment-specific ex vivo sensitivity in OTSCs was concordant with RECIST 1.1-based DCR classification in 7 out of 7 treatment lines (Fig. 5B).

PFS was assessed per therapy line. Across matched therapy lines, 12 evaluable observations from 10 patients were included. Using an EVSS median separation cutoff of 50, median PFS was 141 days (95% CI 76 days to not reached) in the ex vivo resistant group and 445 days (95% CI 305 days to not reached) in the sensitive group (log-rank *p* = 0.0027; Fig. 5C). Continuous EVSS showed exploratory performance for treatment-line-specific PFS (Harrell’s C = 0.731; Somers’ Dxy = 0.462; bootstrap 95% CI 0.024–0.838; *p* = 0.0010; Fig. 5D). In the primary multivariable clustered Cox model adjusted for therapy line including all patients (M0 and M1), higher EVSS was significantly associated with longer PFS (HR per EVSS unit 0.954; 95% CI 0.930–0.979; *p* = 0.00032), corresponding to an HR of 0.625 per 10 EVSS points (95% CI 0.484–0.807, *p* = 0.00032). The therapy line itself was not significantly associated with PFS (HR 0.909; 95% CI 0.567–1.456; *p* = 0.690). Additional adjustment for resection status resulted in only minimal changes of the EVSS effect estimate (HR per 10 EVSS points 0.617 vs 0.625 without adjustment), suggesting no major confounding by resection margin status. Leave-one-out analyses showed consistent effect directions after sequential patient exclusion. Proportional hazards assumptions were not violated (global test *p* = 0.96). Patient #13 was the only patient for whom tissue acquisition occurred after the corresponding matched clinical treatment lines. In an additional temporal sensitivity analysis excluding this patient and both associated treatment-line observations, 10 treatment lines from 9 patients with 6 PFS events remained. The association between higher EVSS and prolonged PFS remained consistent (HR per 10-point increase in EVSS 0.583; 95% CI 0.445–0.763; *p* = 0.00009), indicating that the overall association was not driven by the temporally non-evaluable observations from patient #13.

Sensitivity analyses restricted to exact regimen-level matches showed a consistent overall trend. After exclusion of the three approximate GEM-backbone matches, 9 evaluable treatment lines from 8 patients remained (4 EVSS-resistant, 5 sensitive). Continuous EVSS retained comparable discriminatory performance (Harrell’s C = 0.737; Somers’ Dxy = 0.474; bootstrap 95% CI −0.172 to 1.000; *p* = 0.010). Median PFS was 124 days (95% CI 4 days to not reached) in the resistant group versus not reached (95% CI 305 days to not reached) in the sensitive group (log-rank *p* = 0.023) (Supplementary Fig. S4). In clustered Cox regression, the effect direction remained consistent, although estimates became unstable because of the reduced number of events (HR per 10 EVSS points 0.760; 95% CI 0.416–1.390; *p* = 0.372). After excluding patient #13, the temporally eligible exact-match cohort comprised seven treatment lines from seven patients with three PFS events and retained directionally consistent results (log-rank *p* = 0.012; Harrell’s C = 0.833; Somers’ Dxy = 0.667; bootstrap 95% CI, −0.333 to 1.000). In clustered Cox regression, the effect direction remained consistent, although estimates became unstable because of the reduced number of events (HR per 10 EVSS points: 0.885; 95% CI: 0.483–1.620; *p* = 0.692).

Analyses restricted to non-metastatic patients showed consistent results, with similar discrimination and effect estimates (Harrell’s C = 0.786; Somers’ Dxy = 0.571; bootstrap 95% CI −0.250 to 1.000; log-rank *p* = 0.0039). In clustered Cox analyses, higher EVSS remained associated with prolonged PFS (HR per EVSS unit 0.947; 95% CI 0.922–0.973; *p* = 0.000089), corresponding to an HR of 0.583 per 10 points (95% CI 0.445–0.763), suggesting that the EVSS-PFS association was not driven by metastatic disease (Supplementary Fig. S5).

A Firth-penalized Cox sensitivity analysis showed comparable effect estimates, although statistical significance was attenuated (HR per EVSS unit 0.957; *p* = 0.0686), supporting robustness despite the limited number of events.

Exploratory OS analyses included eight curatively resected patients (5 events, 3 censored), with a median follow-up of 1381 days and median OS of 638 days. Stratified by EVSS, median OS was 434.5 days (95% CI 295 days to not reached) in the ex vivo resistant group and 1107 days (95% CI 638 days to not reached) in the ex vivo sensitive group (log-rank *p* = 0.047) (Fig. 5E). Continuous EVSS showed exploratory discriminatory performance for OS (Harrell’s C = 0.800; Somers’ Dxy = 0.600; bootstrap 95% CI −0.231 to 1.000; *p* = 0.040; Fig. 5F), although confidence intervals remained wide because of the limited sample size. In univariable Cox regression, EVSS showed a consistent effect direction (continuous HR 0.945; 95% CI 0.888–1.006; *p* = 0.076), corresponding to an HR of 0.570 per 10 EVSS points (95% CI 0.306–1.056) The Firth-penalized models confirmed similar estimates (HR per 10 EVSS points 0.625; 95% CI 0.356–1.098; *p* = 0.047), but confidence intervals remained broad.

Together, these exploratory results suggest that EVSSs derived from OTSCs reflect treatment-specific patterns and may capture aspects of individualized treatment sensitivity associated with PFS and OS. While the observed associations were consistent across multiple sensitivity analyses, all survival analyses should nevertheless be interpreted as exploratory and hypothesis-generating given the limited sample size and number of events.

## Discussion

In this study, we established an organotypic tissue slice culture (OTSC) platform from patient-derived PDAC tissue to functionally characterize ex vivo responses to clinically used chemotherapy regimens. The key benefit of OTSCs as patient-derived PDAC models is the close recapitulation of the native, heterogeneous tissue context. By combining cost- and time-efficient OTSC methodology with open-access DIA workflows, we established a reproducible read-out to quantify personalized ex vivo treatment response and explored its association with clinical treatment courses and survival outcomes.

A key strength of OTSCs is the preservation of the native tumor architecture and the complex multicellular TME. Unlike other cultivation techniques, which require tissue dissociation, such as patient-derived cell cultures (PDCCs) or PDOs, OTSCs preserve the spatial organization, stromal composition, and immune-cell populations of the original tissue^29^. Given the pronounced intratumoral heterogeneity of PDAC and its relevance for therapy resistance, preservation of the original tissue architecture represents a key conceptual strength of this model^30–32^. Our histological and digital analyses indicated preservation of the structural tumor architecture as well as stromal and immune cell populations during the cultivation period. Proliferative tumor-cell outgrowth zones at the tissue surface were included in the DIA analyses, as these regions represented viable and Ki67-positive components of the cultured OTSCs rather than preparation artefacts. While relative shifts in immune-cell distribution were observed, the structural tumor-stroma-immune organization remained stable, supporting the suitability of OTSCs as a structurally preserved short-term model for ex vivo pharmacotyping^17–19^. Previous proteomic and transcriptomic analyses in OTSCs demonstrated stable biological signaling pathways during short-term cultivation, supporting preservation of intrinsic tumor biology beyond morphological integrity^33,34^. However, immune functionality was not directly assessed, and the immune compartment in OTSCs remains static without systemic recruitment or priming, limiting conclusions regarding long-term immune interactions^35,36^.

From a feasibility perspective, OTSCs covered the full PDAC stage spectrum and had a high establishment rate (24 out of 27 specimens, 88.9%), exceeding reported rates of 20–80% for PDXs and PDOs^22,37,38^. This represents an advantage over PDX models, which show higher engraftment rates mainly in aggressive, advanced tumors^39,40^. However, in our hands, successful pharmacotyping depended on tissue quality, tumor cellularity, and slice yield. These practical constraints underscore the need for standardized pre-analytical workflows and realistic expectations regarding throughput in a clinical context^24^. Immediate OTSC establishment after resection enabled rapid, individualized assessment of ex vivo treatment response within 8–12 days, including 3–5 days of cultivation and 5–7 days for DIA-based histopathological readout. This timeframe is considerably shorter than that of PDX-based models, which often require 1–3 months before prediction can be assessed, and generally shorter than many PDO-based workflows, although recent PDO platforms have demonstrated increasingly rapid translational timelines^22,38,41^, and aligns with the 6-week window available for adjuvant chemotherapy initiation^42^. Particularly in advanced stage disease rapid assessment is important for informed, personalized treatment decisions. In contrast to PDO- and PDX-based approaches, OTSCs enable pharmacotyping directly within preserved tumor tissue without prior cellular selection or clonal expansion. This allows assessment of treatment response within the native tumor context and within clinically actionable timeframes, positioning OTSCs as a complementary platform for rapid functional stratification rather than a replacement of expansion-based cultivation models.

We developed the EVSS to integrate complementary parameters measuring ex vivo treatment response into a composite response metric. Semi-automated IHC-based DIA enabled fast and standardized quantification of response and calculation of the EVSS, which showed exploratory associations with survival, preoperative CA 19-9 levels and GATA6 expression. The combination of tumor cell content, apoptosis, and proliferation allows integration of these interconnected treatment effects. The EVSS was designed as a biologically integrative composite rather than a mathematically optimized predictive score. While CK7 contributed strongly to the observed signal, individual markers showed heterogeneous and partly unstable performance across exploratory analyses, supporting the use of a composite approach rather than reliance on a single biomarker endpoint. In contrast, resazurin-based metabolic readouts showed weaker and less consistent associations in exploratory analyses, likely reflecting the non-tumor-specific nature of global metabolic activity measurements within stromal-rich PDAC tissue. Thus, the multidimensional EVSS-approach was chosen to capture complementary treatment effects rather than relying on a single biomarker endpoint. However, the EVSS is a cohort-derived composite score, and derivation as well as clinical correlation were performed within the same exploratory cohort, introducing potential cohort-specific overfitting. As EVSS standardization was cohort-dependent and performed across all evaluable samples, recalibration in independent cohorts will be required before broader clinical application. Furthermore, the predefined EVSS cutoff by median separation was used for exploratory subgroup visualization rather than representing a biologically validated threshold. Moderate agreement between automated and visual Ki67 scoring might indicate additional measurement variability within the current EVSS framework. Nevertheless, agreement with manual scoring and biological relevance does not necessarily represent identical measures of analytical performance in heterogeneous tissue architectures^43^. Integration of supervised review could enhance accuracy, as described previously in DIA-focused studies^44^. The exploratory associations between EVSS, CA 19-9, and GATA6 suggest biological consistency between ex vivo drug-response patterns and PDAC disease characteristics. Elevated pre-operative CA 19-9 levels, reflecting tumor burden and aggressive, therapy-resistant tumor phenotype^4,45–47^, were linked to reduced ex vivo sensitivity, whereas higher GATA6 expression, consistent with classical-like subtype biology, was associated with increased treatment responsiveness^6,11,28,44,48^.

The concordance between ex vivo response profiles and clinical outcomes was limited by the highly heterogeneous treatment courses observed across patients in this real-world cohort. Real-world treatment constraints reduced clinical matchability: three patients did not receive systemic therapy due to frailty, while in two additional patients no corresponding ex vivo regimen had yet been incorporated at the respective stage of platform development. Consequently, clinically interpretable treatment-matched pharmacotyping was feasible in 10 of 15 pharmacotyped patients who underwent systemic therapy.

Ex vivo drug exposure revealed pronounced inter-patient heterogeneity in treatment sensitivity, reflecting preserved biological diversity between tumors. The concordance between OTSC responses and RECIST-based DCR in the majority of cases supports the translational relevance of the ex vivo readout. Importantly, exploratory treatment-line-based analyses of matched regimens demonstrated that higher ex vivo sensitivity was associated with longer progression-free survival under the corresponding clinical therapy. Median PFS was 445 days in the ex vivo sensitive group compared with 141 days in the resistant group (log-rank *p* = 0.0027). However, this pronounced Kaplan–Meier separation observed in the resistant subgroup should be interpreted cautiously given the limited number of patients and early clustering of progression events. Continuous EVSS was associated with PFS in exploratory clustered Cox regression (HR per EVSS unit 0.954; 95% CI 0.930–0.979; corresponding to HR 0.625 per 10 EVSS points), with directionally consistent results across sensitivity analyses including therapy line, R-status, and M-status adjustments. Excluding patient #13, the only patient whose tissue was acquired after the matched treatment lines, yielded a comparable EVSS effect estimate, supporting the robustness of the observed PFS association. These treatment-line-specific associations are compatible with a therapy-linked response and suggest that OTSC-derived drug response readouts may reflect treatment-specific vulnerability. However, the present analyses cannot yet distinguish a treatment-specific effect from a general prognostic association. Associations between EVSS and OS across the entire cohort likely reflect a combination of intrinsic tumor biology and treatment sensitivity patterns; median OS was 1107 days in ex vivo sensitive patients versus 434.5 days in resistant patients (log-rank *p* = 0.047), while Cox models indicated a consistent effect direction.

Experimental non-translational studies previously demonstrated time- and dose-dependent treatment effects across pharmacological agents, including cytotoxic drugs and pathway inhibitors, supporting the suitability of this model for treatment response assessment^24,34^. The biological rationale underlying OTSC-based pharmacotyping lies in the preservation of tumor architecture and cell-cell interactions that influence treatment response. However, static drug exposure does not replicate intratumoral pharmacokinetics or systemic effects, including tissue penetration and spatial drug concentration gradients within the PDAC microenvironment. OTSCs represent a complementary rather than comprehensive model of in *vivo* tumor biology. Further, several limitations should be considered. The study cohort was limited in size and heterogeneous with regard to disease stage and treatment history, precluding larger multivariable adjustment. The matched survival analyses were based on a limited number of patient clusters, which may reduce inferential precision despite complementary sensitivity analyses, although effect estimates remained directionally consistent across models. The wide confidence intervals observed across survival analyses further underscore the statistical limitations imposed by the cohort size. Treatment matching partly relied on therapeutic backbone approximation in two patients (#4, #5) in whom GnP was administered clinically whereas GEM was tested ex vivo. This reflected the developmental-stage implementation of the OTSC pharmacotyping platform, during which drug regimens were introduced sequentially, resulting in partially overlapping treatment-testing cohorts. Consequently, the present study should be interpreted as an exploratory translational feasibility study rather than a definitive treatment-selection trial. The present data are not yet intended to support direct clinical treatment selection. Furthermore, OTSCs were predominantly derived from primary tumors, whereas clinical outcomes often reflect systemic disease progression, where potential chemosensitivity discordance between primary and metastatic sites may occur. Finally, OTSCs are inherently limited to short-term cultivation and depend on high-quality fresh tissue, which may affect scalability and standardization across centers^24,49,50^.

Future research should focus on prospective validation in larger, regimen-stratified cohorts with predefined EVSS thresholds, standardized workflows, and integration of molecular subtyping, spatial OMICs, and multicenter reproducibility testing.

In conclusion, OTSCs preserve the individual tumor tissue architecture in a time- and cost-effective ex vivo model, including the structural (immunological) TME. The present proof-of-concept study provides translational evidence that functional treatment response profiling in preserved PDAC tissue is feasible and supports further prospective evaluation as a rapid functional pharmacotyping platform. Compared with static biomarker approaches, OTSC-based testing captures dynamic treatment responses within the native tumor context and may complement molecular stratification strategies. OTSCs may help bridge the gap between experimental modeling and individualized treatment approaches and support further development toward clinically applicable response-guided oncology; however, further evaluation of this approach in larger, independent clinical studies is warranted.

## Methods

### Patient selection and study design

Fresh PDAC specimens were obtained from patients undergoing pancreatic resection between 2020 and 2024 at the Department of Surgery of the University Medical Center Schleswig-Holstein, Campus Lübeck (Fig. 1A). Eligible patients were ≥18 years, provided written consent, and had suspected PDAC of any stage. The study was designed as a translational, exploratory feasibility study with three predefined objectives: (i) establishment and quality-controlled implementation of organotypic tumor slice culture (OTSC) generation and short-term cultivation, (ii) quantitative assessment of ex vivo drug sensitivity of clinically used chemotherapy regimens, and (iii) evaluation of associations between ex vivo drug sensitivity and clinicopathological as well as outcome parameters. Analysis sets were defined a priori and comprised (i) the OTSC establishment cohort (*n* = 27), (ii) the pharmacotyping cohort (*n* = 15), and (iii) the clinical correlation cohort (*n* = 10) (Fig. 1B & Table 1). The design followed REMARK recommendations for biomarker studies^51^. During the study period, OTSC cultivation and pharmacotyping workflows were established iteratively, with early cases used for workflow optimization before regimen-specific drug testing was implemented. Drug regimens were introduced sequentially over time, resulting in partially overlapping treatment-testing cohorts.

### Ethics approval and consent to participate

The study was approved by the ethics committee of the University of Lübeck (#16-281) in accordance with the Declaration of Helsinki. All patients included provided written consent.

### Tissue preparation, sectioning and cultivation

After pathological evaluation (Table 1 & Supplementary Table S1), tumor tissue was transferred in MACS Tissue Storage Solution (Miltenyi Biotec, Bergisch-Gladbach, Germany) on ice, trimmed (~0.5 cm^3^), stabilized in low-melt agarose (Roth Chemie GmbH, Karlsruhe, Germany) and sectioned into 300 µm slices using a vibratome Leica VT1200 S (Leica Biosystems, Nussloch GmbH, Germany). Slices were placed on semiporous Polytetrafluoroethylene (PTFE) culture inserts (Millipore, Darmstadt, Germany) in 6-well plates with supplemented DMEM/F12 and incubated at 37 °C/5% CO_2_ for 72–120 h. Medium was changed every 24 h. Tissue viability was assessed daily by resazurin reduction (resazurin sodium salt, Sigma Aldrich, Steinheim, Germany) as described elsewhere^20^.

### Ex vivo drug treatment

Drug exposure was performed at 24, 48, 72 and 96 h via medium supplementation. Treatment schemes applied were: GEM monotherapy (GEM: 89.3 µM (GRY, Rammelsbach, Germany)), GnP (GEM: 89.3 µM + albumin-bound paclitaxel (nab-paclitaxel): 4.27 µM (Ratiopharm, Ulm, Germany) and FFX (5-FU: 426 µM (MEDAC, Wedel, Germany), oxaliplatin: 4.96 µM (GRY), irinotecan: 5.78 µM (MEDAC, Wedel, Germany), leucovorin: 6.18 µM (GRY). Concentrations of clinical standard regimens GEM, GnP and FFX were selected based on clinically relevant peak plasma concentrations in vivo (C_max_)^25^ and previous ex vivo studies^18,26,27^. To account for intratumoral heterogeneity, OTSCs from each patient were processed in duplicates with treated slices paired to adjacent untreated controls.

### Histological and immunohistochemical analysis

OTSCs were fixed at 0, 72 and 120 h, paraffin-embedded, sectioned (4 µm), and stained by hematoxylin & eosin (H&E) and IHC as described previously^20^. Our standard panel of histopathological markers for tissue structure and viability included H&E, cytokeratin 7 (CK7), Ki67 and cleaved caspase 3 (CC3). The panel was extended by pan-cytokeratin (PanCK), α-SMA, Sirius Red, CD3, CD4, CD8, CD68 and CD163 for stromal and immune cell populations in selected samples for TME characterization. GATA6 expression was stained to assess the PDAC subtype. Supplementary Table S4: Primary antibodies, dilution and manufacturer information.

### Quality control and histopathological evaluation

All OTSCs underwent histological quality control based on H&E staining. Control OTSCs were reviewed by a specialized pathologist (CK). Specimens with insufficient tumor cellularity were excluded from treatment response and clinical correlation analyses. In one case (patient #14), the FFX-treated slice was lost during embedding, whereas the GnP-treated slice and corresponding controls were included in the analyses.

### Machine learning-based DIA

Slides were scanned as virtual whole-slide-images (WSIs) (3DHistech Panoramic Desk 1.14 slide scanner, 3DHistech, Budapest, Hungary). We assessed morphological parameters semi-automatically using the open-access DIA software QuPath (Fig. 2A)^23^. Following the preprocessing steps (1) *estimating stain vectors*, automatic tissue detection based on hematoxylin background stain, and manual removal of artefacts, the built-in *(positive) cell detection* command was used to quantify cells (2). For immunohistochemically stained slides, DAB intensity of cells was quantified. Additional texture and smoothing features for morphological cell cluster/area characterization were calculated (3). Distinct tissue components, i.e., tumor, stroma, necrosis, apoptosis, fibrosis and immune cells, were manually annotated to train staining-specific random-forest classifiers (4). We established the DIA workflow using a predefined, (i) training (*n* = 64 WSIs), (ii) validation (Ki67 *n* = 108 WSIs, CC3 *n* = 103 WSIs) and (iii) test (*n* = 137 WSIs for Ki67, CC3 respectively) set. Overall, classifier training comprised 1196 manually annotated regions across 64 WSIs (Ki67: 344 annotations across 21 WSIs; CC3: 439 annotations across 22 WSIs; H&E: 413 annotations across 21 WSIs). Tumor annotations used for classifier training were guided by virtual alignment with consecutive CK7-stained sections to improve robustness of tumor identification and were additionally verified by visual inspection. Annotated tissue regions were subdivided into standardized 2500 µm² quantification areas. Only areas containing more than 100 detected cells were included to ensure robust percentage-based assessment of Ki67 and CC3 positivity, and median values across all evaluable areas per WSI were used for downstream analyses. The workflow steps were integrated via *groovy* scripts to enable high-throughput batch processing and were applied to approximately 250 WSIs per staining across the establishment feasibility and pharmacotyping cohorts, including sections that were later excluded during histopathological cohort refinement. Following classifier development, the workflow was applied to 137 quantified OTSC sections across multiple stainings and treatment conditions of the pharmacotyping cohort (Supplementary Fig. S6A). A median of 2 OTSC sections per condition were analyzed, corresponding to a median of 28 quantified tissue regions per condition. In addition, two workflows quantified immune-tumor interactions: (1) spatial distance of immune cells to nearest tumor cell; (2) immune infiltration into tumor regions (Fig. 2B). For detailed standard operating procedure, representative QuPath workflow screenshots, and corresponding Groovy scripts, see: Supplementary Protocol S1. Standard operating procedure for machine-learning-supported digital image analysis of PDAC organotypic tissue slice cultures in QuPath; Supplementary Figs. S7–S13; Supplementary Code S1. QuPath/Groovy scripts for machine-learning-supported digital image analysis of PDAC-OTSCs) .

### Validation of classification workflow

Ki67 and CC3 percentages in tumor-classified regions were evaluated by blinded visual scoring. Visual score thresholds were defined as follows: Ki67 (0–3%, 3–20%, >20%) and CC3 (0-33%, 33-66%, >66%). Agreement assessed by Cohen’s κ using GraphPad’s QuickCalcs^52^, yielding κ = 0.536 and weighted κ = 0.590 for Ki67 (79/108 sections, 73.15%) and κ = 0.724 and weighted κ = 0.772 for CC3 (85/103 sections, 82.52%). Moderate agreement for Ki67 assessment has been reported previously in both visual and digital pathology studies, reflecting the inherent variability of semi-quantitative IHC interpretation in heterogeneous tissue architectures and narrow ordinal cutoff intervals^53,54^. In addition, classifier outputs were visually reviewed using QuPath’s classification-specific color masks to assess plausibility and identify potential misclassifications. See Supplementary Fig. S6B, C.

### Ex vivo drug sensitivity score (EVSS)

Ex vivo treatment effects were quantified by an ex vivo sensitivity score (EVSS). Three parameters were combined: CK7 positive cell fraction, CC3 H-score in tumor-classified cells, and Ki67 positive tumor cell fraction. CC3 was quantified using an H-score because both staining intensity and the proportion of positive tumor cells were considered biologically informative for apoptotic activation^55^. The CC3 H-score was calculated by assigning tumor cells to weak, moderate, or strong staining categories, multiplying the percentage of cells in each category by 1, 2, or 3, respectively, and summing the results to yield a score ranging from 0 to 300. Signs of CK7 and Ki67 were inverted so that reduced expression indicated increased drug sensitivity. Treatment-induced changes were defined in Eqs. (1), (2) and (3):

1 $${\Delta }_{{CK}7}={CK}{7}_{\mathrm{treated}}-{CK}{7}_{\mathrm{untreated}}$$

2 $${\Delta }_{{CC}3}={CC}{3}_{\mathrm{treated}}-{CC}{3}_{\mathrm{untreated}}$$

3 $${\Delta }_{{Ki}67}={Ki}{67}_{\mathrm{treated}}-{Ki}{67}_{\mathrm{untreated}}$$

For each marker, values of treated slices were (i) normalized to matched untreated controls and (ii) subsequently Z-standardized according to Eq. (4):

4 $$Z({x}_{i})=\frac{{x}_{i}-{\mu }_{x}}{{\sigma }_{x}}$$

where ${\mu }_{x}$ and ${\sigma }_{x}$ were calculated across all evaluable pharmacotyping samples rather than within individual treatment regimens to generate a unified exploratory response metric. Let *i* denote the patient index. The raw EVSS was calculated as the mean of standardized parameter changes according to Eq. (5):

5 $${EVS}{S}_{\mathrm{raw}},{i}=\frac{1}{3}\left[-\,Z\left({\Delta }_{{CK}7,i}\right)+Z\left({\Delta }_{{CC}3,i}\right)-Z\left({\Delta }_{{Ki}67,i}\right)\right]$$

Equal weighting was chosen a priori to avoid overfitting in the present exploratory cohort. The final EVSS was obtained by mapping pooled raw scores to a rank-based percentile scale for visualization according to Eq. (6), as shown in Fig. 2C:

6 $${EVS}{S}_{i}=100\cdot \frac{{r}_{i}-0.5}{N}$$

where ${r}_{i}$ is the rank of ${{EVS}S}_{\mathrm{raw},i}$ within the pooled distribution and N is the number of pooled values.

Exploratory analyses of individual EVSS components demonstrated heterogeneous performance across biomarkers. CK7 and Ki67 showed partially consistent associations with survival parameters, whereas CC3 and resazurin demonstrated weaker or less stable associations across discrimination and Cox-based metrics. Overall, the composite EVSS showed comparatively stable performance across discrimination, hazard-ratio, and sensitivity analyses (Supplementary Fig. S2).

### Correlation with clinicopathological parameters

Clinical courses were assessed by evaluation of radiological imaging reports, carbohydrate antigen 19-9 (CA 19-9) levels, time of patient death, and timepoints of chemotherapy administration from institutional records and external physician correspondence. Progression-free survival (PFS) was the primary endpoint and defined per therapy line from treatment initiation to radiological progression (RECIST 1.1^56^) or death, whichever occurred first, within the same treatment line. The observation period was restricted to the respective therapy line; events after initiation of a subsequent therapy were not attributed to the preceding line. Patients without an event were censored at last follow-up, last imaging, or immediately before the start of a subsequent therapy. The disease control rate (DCR) per therapy line was defined by the first radiological report after therapy initiation. Cases without formal post-treatment restaging imaging or with post-treatment tissue acquisition were excluded from DCR concordance analysis. OS was defined from surgical resection to death and was restricted to non-metastatic patients. Clinical matching was performed based on therapies received during longitudinal follow-up. Matches were classified as exact regimen-level matches, approximate pharmacologic backbone matches, or non-comparable matches (Supplementary Table S3).

### Statistical analysis

IHC data were log-transformed before parametric testing. Repeated-measures ANOVA or mixed-effects ANOVA were applied depending on data completeness. EVSS was either evaluated as a continuous variable or dichotomized at the cohort median (≥50 vs <50). Exploratory baseline comparisons were performed using the Mann–Whitney U test for continuous variables and Fisher’s exact test for categorical variables with exploratory binary grouping where appropriate. Continuous variables are reported as median and range, whereas categorical variables are presented as absolute counts and percentages. Associations with clinicopathological markers (CA 19-9, GATA6) were analyzed using Spearman correlation and linear regression. Kaplan-Meier curves were generated for dichotomized EVSS groups and compared using log-rank tests. Time-to-event endpoints were analyzed per therapy-line (PFS) or patient level (OS). Discriminatory performance for time-to-event outcomes was assessed using Harrell’s C-index and Somers’ D with bootstrap confidence intervals (5000 iterations). Correlations between EVSS and survival outcomes were evaluated using Cox proportional hazards regression. Clustered Cox models with robust variance estimation accounted for intra-patient correlation per therapy-line (cluster variable: patient ID). Given the limited number of patient clusters, inferential estimates were interpreted cautiously. Covariates included EVSS, therapy line, and resection status with stratification by metastatic status where applicable. A temporal sensitivity analysis excluded patient #13, whose tissue had been acquired after the matched treatment lines; the same restriction was applied to the exact-match cohort. OS analyses were conducted using Cox models per patient. Proportional hazards assumptions were tested using Schoenfeld residuals. Robustness of the EVSS-PFS association was examined using leave-one-out sensitivity analyses with sequential patient exclusion. Penalized Cox regression with Firth correction was subsequently performed as a sensitivity analysis following the primary Cox models to address small-sample bias and model instability. Analyses were performed in GraphPad Prism v10.3.2, R v4.4.1, RStudio v2026.01.0, and Jamovi v2.6.44.

## Supplementary information

Supplementary information

## Data Availability

All data generated or analyzed during this study are included in this published article and its supplementary information files.

## Code availability

Code for digital pathology is available in the Supplementary Information files of this manuscript (Supplementary Code S1. QuPath/Groovy scripts for machine-learning-supported digital image analysis of PDAC-OTSCs).
