# Supplementary material for: Tumor slice cultures enable rapid ex vivo profiling of patient-specific drug sensitivity in pancreatic ductal adenocarcinoma
Source: NPJ Precis Oncol. 2026 Aug 20;10:325. doi: 10.1038/s41698-026-01663-z (PMC13494009; doi:10.1038/s41698-026-01663-z)
Supplement: Supplementary file 1 [file 41698_2026_1663_MOESM1_ESM.pdf]

Figures

Figure S1: Exploratory correlations between preoperative CA19-9 levels and baseline GATA6 expression across treatment cohorts.

Figure S2: Exploratory comparison of EVSS and individual biomarker components for treatment-line-specific PFS.

Figure S3: Clinical courses of patients #1-#18.

Figure S4: Clinical correlation of treatment line specific PFS in patients with exact regimen matches only.

Figure S5: Clinical correlation of treatment line specific PFS in non-metastasized patients.

Figure S6: DIA and visual scoring interrater reliability.

Figure S7: Representative QuPath tissue detection settings illustrating Protocol S1.

Figure S8: Representative QuPath cell detection and intensity feature calculation workflow illustrating Protocol S1.

Figure S9: Representative QuPath cell detection and intensity feature calculation settings illustrating Protocol S1.

Figure S10: Representative QuPath object-classification training workflow illustrating Protocol S1.

Figure S11: Representative QuPath classifier settings illustrating Protocol S1.

Figure S12: Representative QuPath data export workflow illustrating Protocol S1.

Figure S13: Representative QuPath batch-processing workflow illustrating Protocol S1.

Tables

Table S1: Clinicopathological characteristics.

Table S2: OTSC response and treatment line specific PFS sorted by applied treatment option ex vivo.

Table S3. Patient-level and treatment-line-level overview of OTSC pharmacotyping cases.

Table S4: Primary antibodies, dilution and manufacturer information.

Protocol and Code

Protocol S1: Standard operating procedure for machine-learning-supported digital image analysis of PDAC organotypic tissue slice cultures in QuPath.

Code S1: QuPath/Groovy scripts for machine-learning-supported digital image analysis of PDAC-OTSCs.

Figure S1

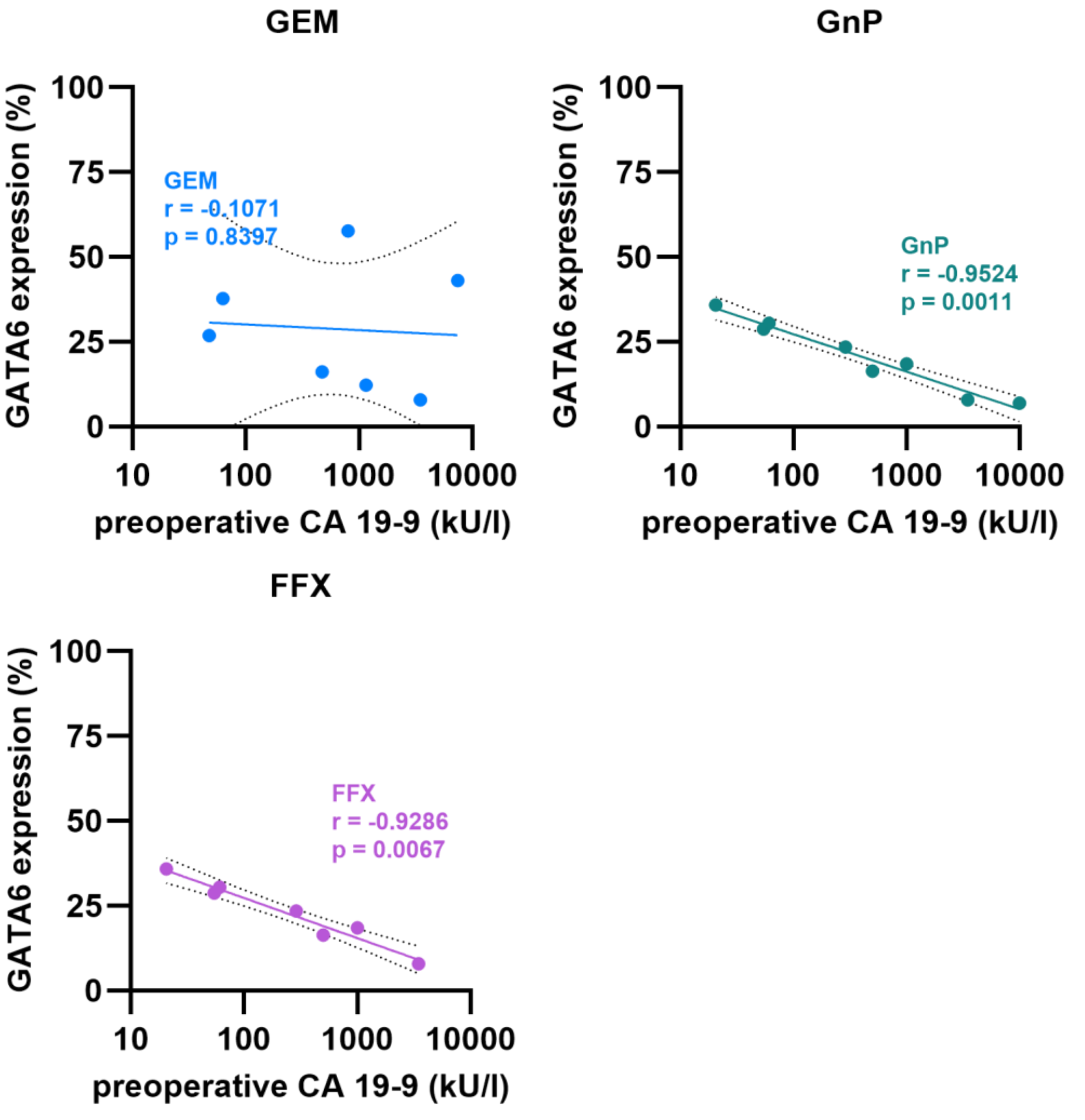

Figure S1: Exploratory correlations between preoperative CA19-9 levels and baseline GATA6 expression across treatment cohorts.

Figure S2

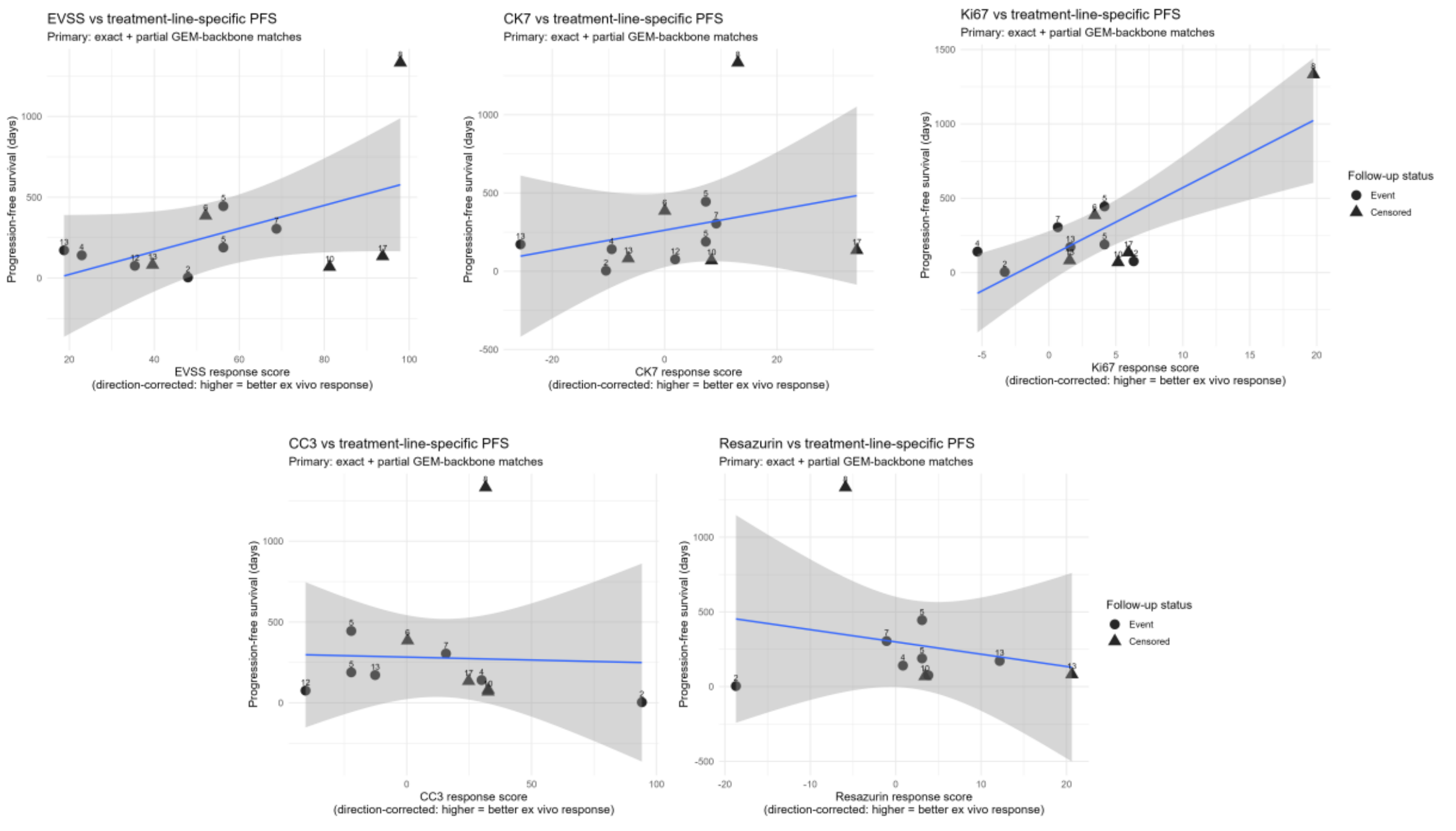

Marker	C-index	Somers' D (95% CI)	HR per 1 SD (95% CI)	Cox p
**EVSS**	0.731	0.462 (0.02 to 0.85)	0.30 (0.16-0.58)	0.0003
**CK7**	0.756	0.513 (-0.03 to 0.93)	0.45 (0.07-2.82)	0.395
**Ki67**	0.679	0.359 (-0.44 to 1.00)	0.21 (0.05-0.94)	0.041
**CC3**	0.474	-0.051 (-0.82 to 0.81)	1.73 (0.37-8.19)	0.490
**Resazurin**	0.500	0.000 (-0.80 to 0.74)	0.28 (0.12-0.64)	0.0027

Figure S2. Exploratory comparison of EVSS and individual biomarker components for treatment-line-specific PFS. **(A)** Scatterplots of EVSS, CK7, Ki67, CC3, and resazurin response scores versus treatment-line-specific PFS in the primary cohort (exact + partial GEM-backbone matches). **(B)** Summary of exploratory discrimination and Cox regression metrics for EVSS and individual biomarkers.

Figure S3

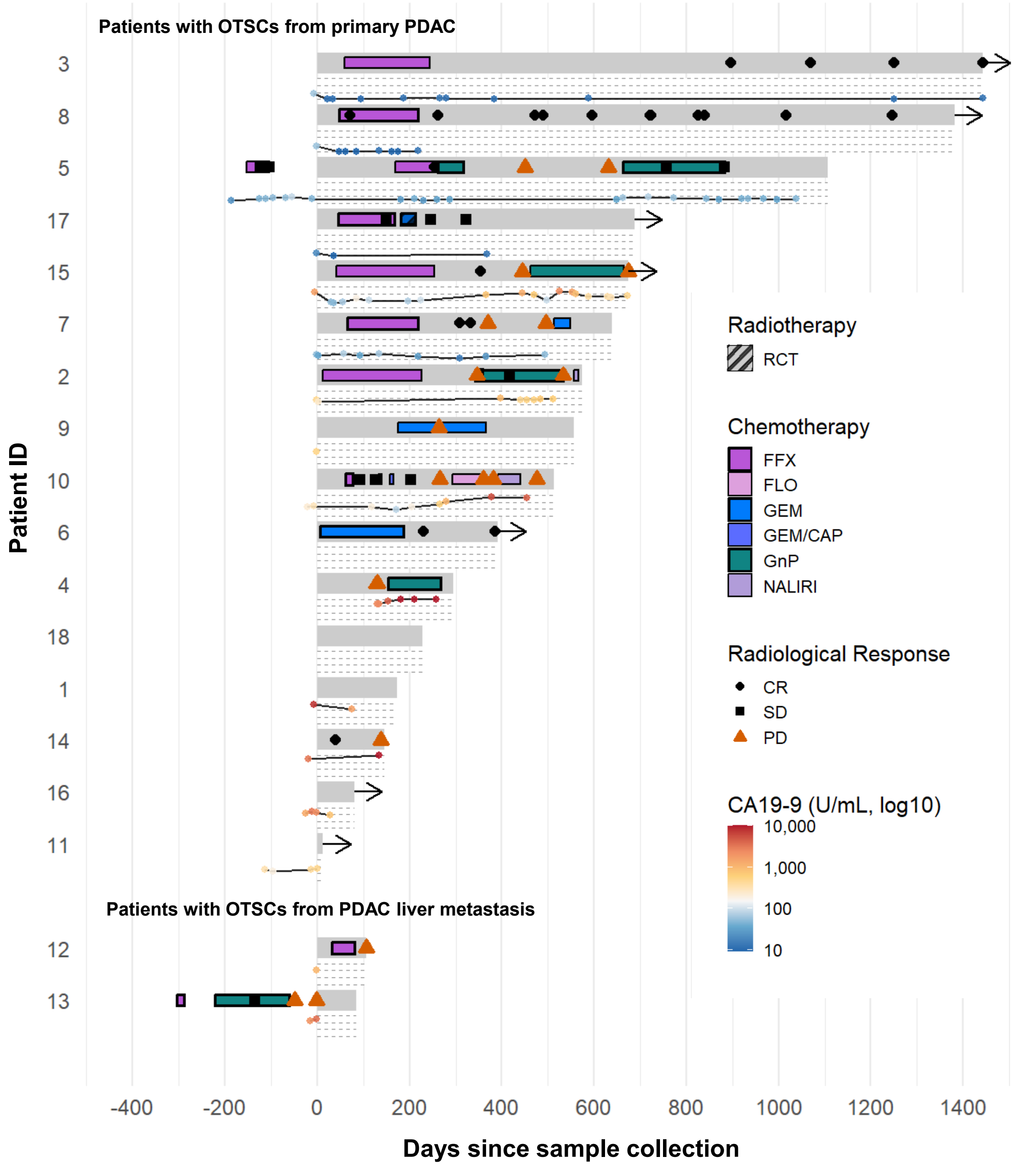

Figure S3: Clinical courses of patients #1-#18. Clinical courses of all patients with *ex vivo* drug response tested in OTSCs. Upper panel: primary PDAC (M0); lower panel: liver metastases (M1). Grey bars indicate overall survival after specimen collection (day 0). Colored bars represent administered chemotherapy (FFX, FLO, GEM, GEM/CAP, GnP, NALIRI). Radiological responses (CR: complete response, SD: stable disease, PD: progressive disease) and log10-transformed CA19-9 levels are shown. Arrows indicate censored observations. Courses of patients #11, #15 and #16 are shown as OTSCs were treated ex vivo but were excluded later for ex vivo drug response analysis as they did not present with adequate tumor cell content. RCT, radiochemotherapy.

Figure S4

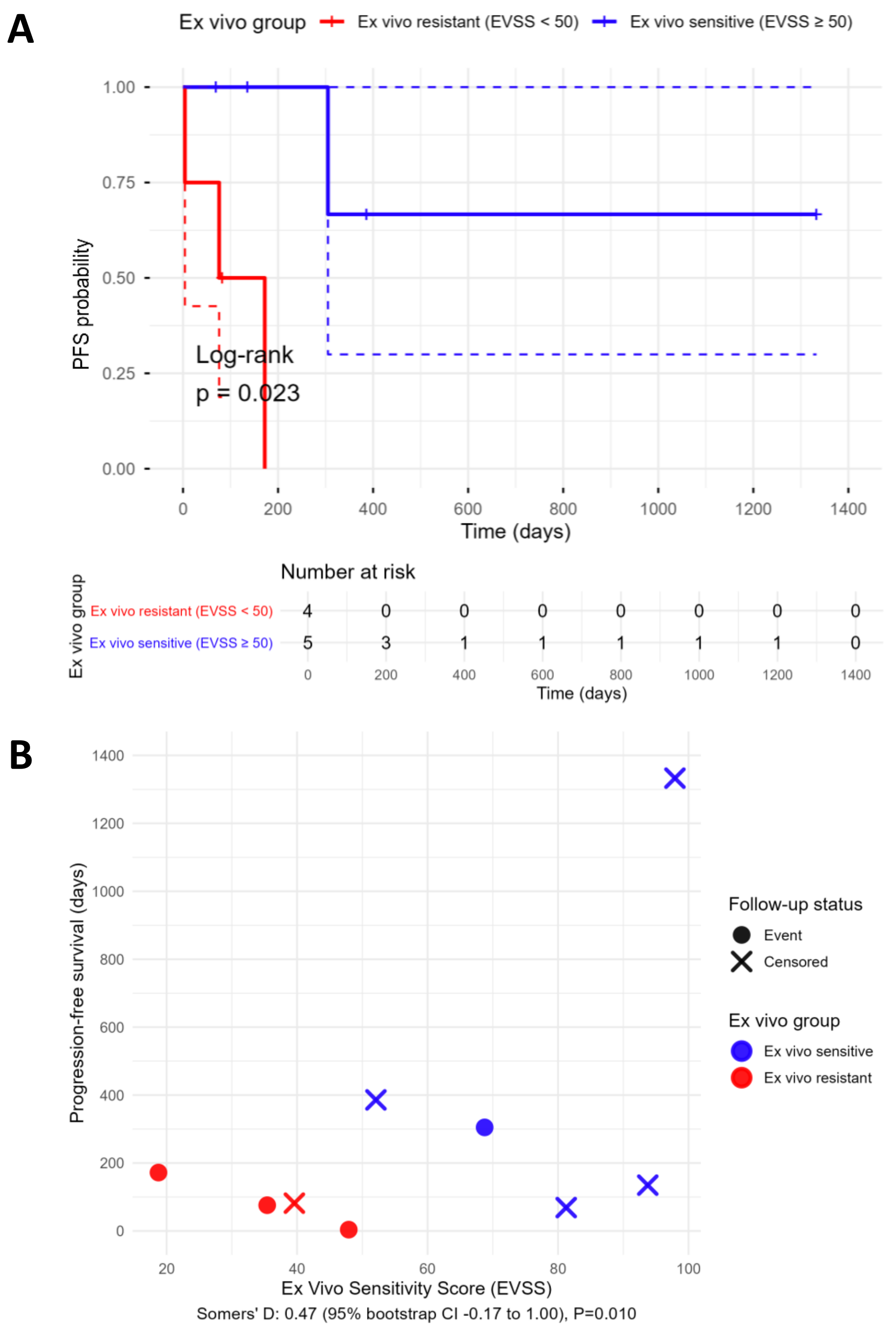

Figure S4: Clinical correlation of treatment line specific PFS in patients with exact regimen matches only. (A) Kaplan-Meier analysis of treatment-specific progression-free survival (PFS) stratified by *ex vivo* sensitivity (EVSS ≥ 50 vs < 50) in n=9 treatment lines of 8 patients. Log-rank test. (B) Correlation of EVSS with PFS shown as Somers' D with bootstrap confidence intervals.

Figure S5

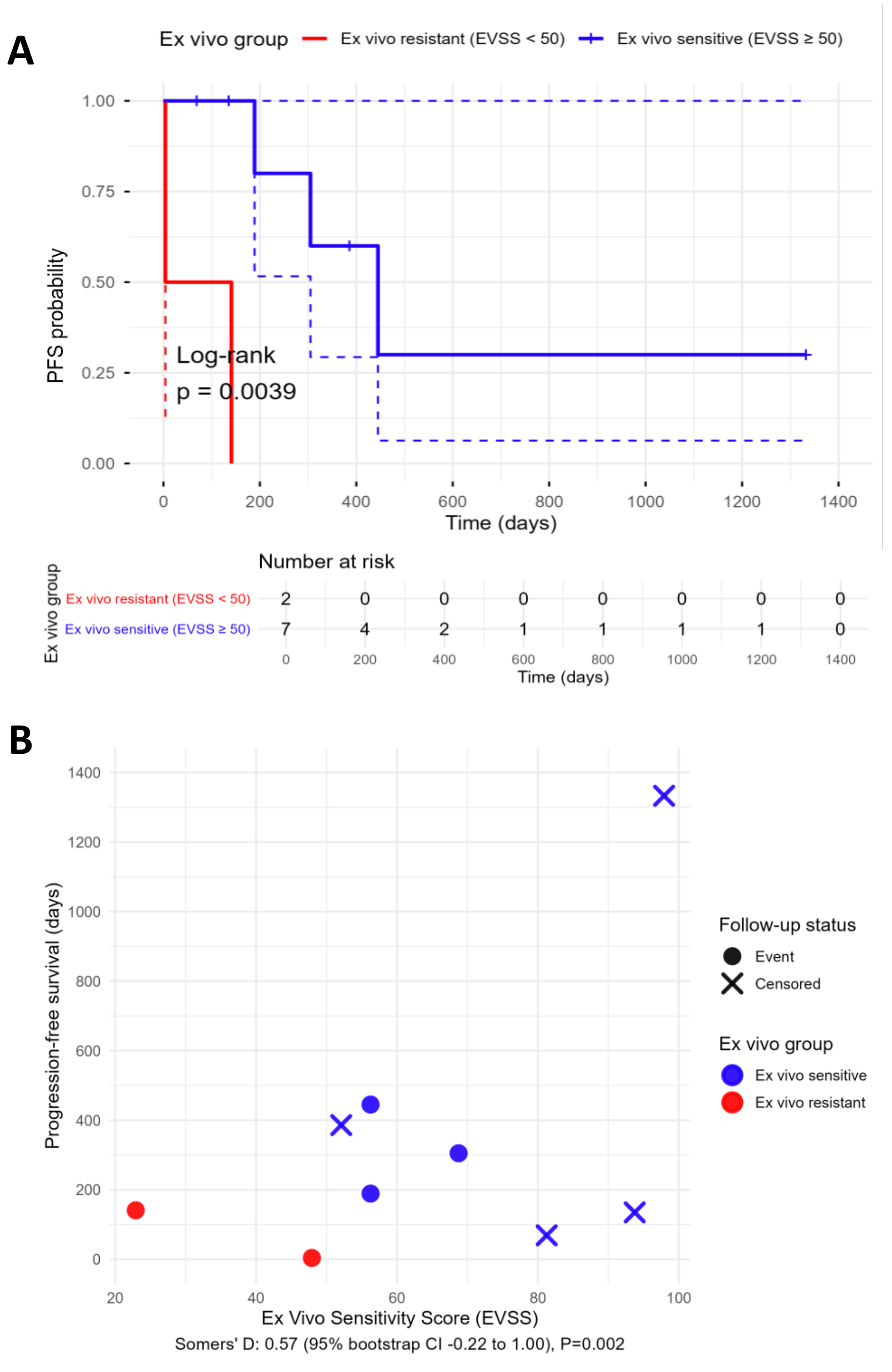

Figure S5: Clinical correlation of treatment line specific PFS in non-metastasized patients (M0). (A) Kaplan-Meier analysis of treatment-specific progression-free survival (PFS; M0) stratified by *ex vivo* sensitivity (EVSS ≥ 50 vs < 50) in n=9 treatment lines of 8 patients. Log-rank test. (B) Correlation of EVSS with PFS shown as Somers' D with bootstrap confidence intervals.

Figure S6

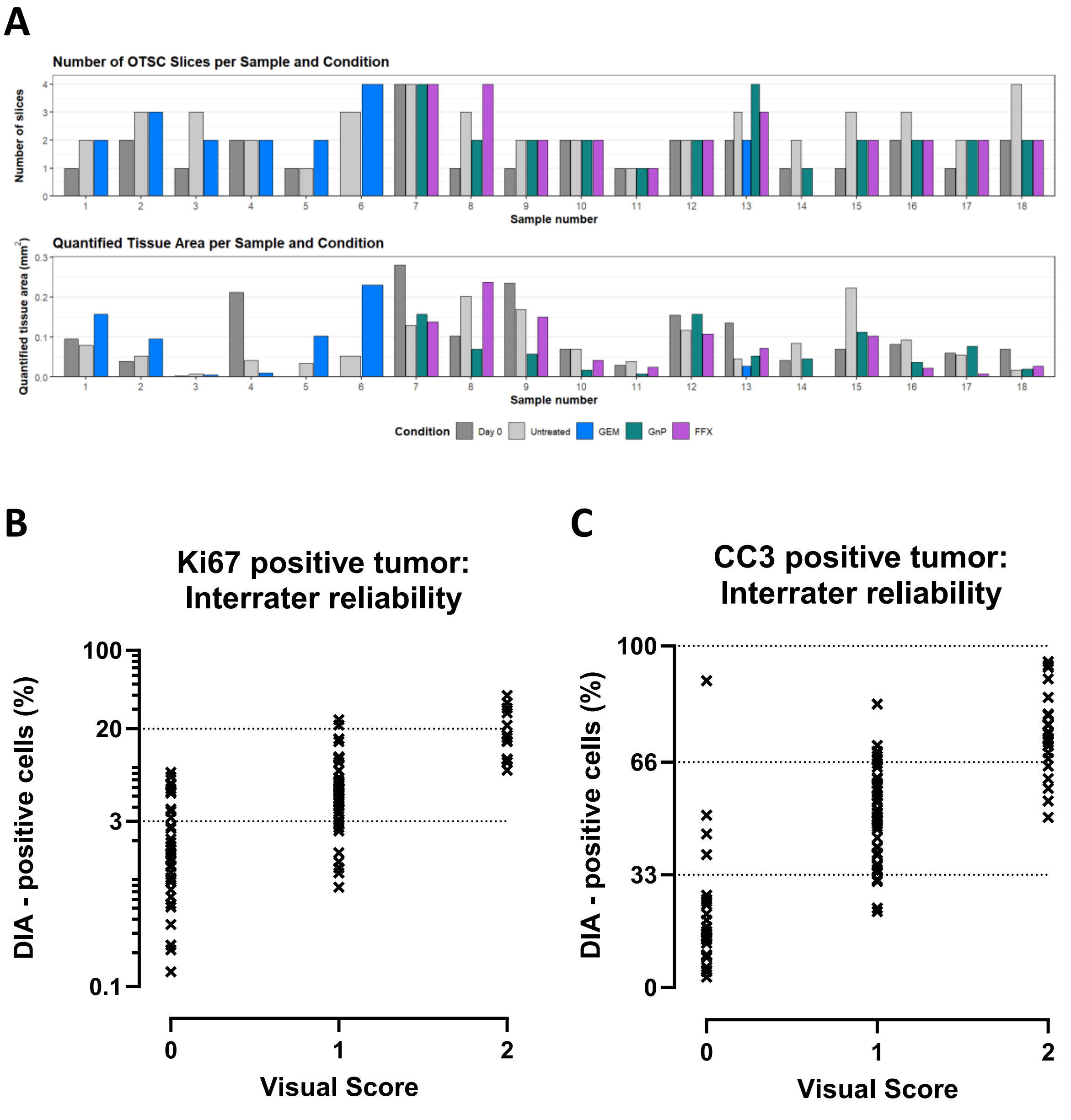

Figure S6: DIA and visual scoring interrater reliability. (A) Biological replication and spatial sampling depth of the standardized IHC-based *ex vivo* readout. Upper panel: number of analyzed OTSC sections per sample and treatment condition. Lower panel: corresponding quantified tissue area derived from summed 2500 µm² image-analysis regions used for QuPath-based digital image analysis. Samples later excluded from clinical correlation analyses because of insufficient tumor cellularity (#11, #15, #16) are included. **(B)** Interrater reliability of DIA and visual assessment of Ki67 positive tumor cells. Digital index was assessed on a percentage scale, sorted in intervals from 0-3%,3-20% and 20 to 100% in line with regular Ki67 index scoring. Visual scoring was done on an ordinal scale, where a score of 0 indicated a Ki67 tumor percentage of 0-3% (low), 1 a percentage of 3-20% (intermediate) and 3 a percentage between 20-100% (high). **(C)** Interrater reliability of DIA and visual assessment of CC3 positive tumor cells. Digital index was assessed on a percentage scale, sorted in intervals from 0-33%,33-66% and 66-100% in line with regular Ki67 index scoring. Visual scoring was done on an ordinal scale, where a score of 0 indicated a Ki67 tumor percentage of 0-33% (low), 1 a percentage of 33-66% (intermediate) and 3 a percentage between 66-100% (high).

Table S1

A

Patient	Sex	Age	T	N (detail)	M	R	AJCC 8	G	GATA6 day 0 (%)	PreOp CA 19-9 (kU/l)	Survival status	OS (days)
1	F	72	T3	N2 (4/52)	M0	R1	III	G3	43.03	7416	Deceased	173
2	F	64	T3	N2 (6/36)	M0	R0	III	G3	16.18	470	Deceased	574
3	M	75	T3	N0 (0/65)	M0	R0	IIA	G3	37.74	62.4	ALIVE	1442
4	M	73	T2	N1 (3/27)	M0	R0	IIB	G3	12.27	1152.00	Deceased	295
5	F	72	ypT2	ypN0 (0/18)	M0	R1	IB	–	24.58	47.70	Deceased	1107
6	F	86	T2	N1 (1/25)	M0	R0	IIB	G2	57.67	796.00	ALIVE	392
7	M	55	T2	N1 (2/30)	M0	R0	IIB	G2	22.80	54.30	Deceased	638
8	M	58	T2	N2 (4/33)	M0	R0	III	G2	30.43	60.8	ALIVE	1381
9	M	72	T3	N1 (1/9)	M0	R0	IIB	G3	16.32	499	Deceased	557
10	F	68	T2	N1 (3/15)	M0	R1	IIB	G3	23.43	289	Deceased	514
12	M	75	Tx	Nx	M1	NA	IV	–	16.32	1001	Deceased	107
13	M	57	Tx	Nx	M1	NA	IV	–	7.84	3474.00	Deceased	84
14	M	74	T3	N0 (0/13)	M0	R0	IIA	G2	6.95	3051.00	Deceased	145
17	M	58	T2	N2 (9/18)	M0	R0	III	G3	35.81	20.6	ALIVE	667
18	F	78	T3	N2 (8/29)	M0	R1	III	G3	12.49	NA	Deceased	229

Table S1: Clinicopathological characteristics. (A) Baseline characteristics, pathological characteristics, biomarkers and overall survival in patients with *ex vivo* drug response tested in OTSCs. Tumor stage according to 8th AJCC/UICC classfication.

Table S2

Patient/ *ex vivo* treatment	clinical chemotherapy lines	matching treatment line	PFS (days) for matched treatment line	PFS status for matched treatment line (Event/Censored)	PFS Event type	matching treatment option *ex vivo*	EVSS (rank-based)	CK7 % difference	Ki67 % difference	CC3 H-Score difference
***ex vivo* GEM**										
1	none	–	–	–	–	–	14.58	9.48	-1.05	-85.10
2	1. FFX, 2. GEM, 3. GnP	GEM (2.)	4	Event	PD	GEM	47.92	10.43	3.30	94.18
3	FFX	–	–	–	–	–	60.42	-5.16	-1.39	6.39
4	GnP	GnP	141	Event	Death	GEM	22.92	9.41	5.34	30.00
5	1. FFX, 2. GnP, 3. GnP	GnP (2.)	189	Event	PD	GEM	56.25	-7.34	-4.15	-22.21
5	"	GnP (3.)	445	Event	Death	GEM	"	"	"	"
6	GEM	GEM	386	Censored	–	GEM	52.08	-0.04	-3.42	0.36
13	1. FFX, 2. GnP	–	–	–	–	–	6.25	25.30	12.91	91.15
***ex vivo* GnP**										
7	1. FFX, 2. GeM	–	–	–	–	–	64.58	-14.36	-0.56	-10.48
8	FFX	–	–	–	–	–	85.42	-5.79	-6.12	38.33
9	GEM	–	–	–	–	–	72.92	0.22	-2.94	50.24
10	1. FFX, 2. GEM/CAP, 3. FLO, 4. NALIRI	–	–	–	–	–	77.08	-7.94	-2.05	86.00
12	FFX	–	–	–	–	–	10.42	7.32	8.73	-13.31
13	1. FFX, 2. GnP	GnP (2.)	172	Event	PD	GnP	18.75	25.64	-1.60	-12.65
14	none	–	–	–	–	–	2.08	-0.59	11.17	-55.99
17	FFX	–	–	–	–	–	89.58	-30.43	3.14	51.70
18	none	–	–	–	–	–	31.25	5.42	-1.05	-3.63
***ex vivo* FFX**										
7	1. FFX, 2. GEM	FFX (1.)	305	Event	PD	FFX	68.75	-9.19	-0.66	15.71
8	FFX	FFX (1.)	1333	Censored	–	FFX	97.92	-13.04	-19.75	31.59
9	GEM	–	–	–	–	–	43.75	0.50	-3.41	12.75
10	1. FFX, 2. GEM/CAP, 3. FLO, 4. NALIRI	FFX (1.)	69	Censored	–	FFX	81.25	-8.35	-5.16	32.58
12	FFX	FFX (1.)	76	Event	PD	FFX	35.42	-1.88	-6.33	-40.51
13	1. FFX, 2. GnP	FFX (1.)	82	Censored	–	FFX	39.58	6.47	-1.55	32.63
17	FFX	FFX (1.)	135	Censored	–	FFX	93.75	-34.20	-5.94	24.79
18	none	–	–	–	–	–	27.08	-1.98	2.39	-24.24

Treatment line matching *ex vivo* tested drug: orange *Ex vivo* sensitive: blue *Ex vivo* resistant: red

Table S2: OTSC response and treatment line specific PFS sorted by applied treatment option *ex vivo*. Treatment lines with matching treatment option *ex vivo* are marked in orange. *Ex vivo* response parameters are color-sorted (blue: sensitive, red: resistant).

Table S3

StudyID	Sample source	Timing of tissue acquisition relative to systemic therapy	Tissue collected before/after matched line	Ex vivo regimen	Clinical regimen	Match class	Inclusion/exclusion rationale
**1**	Primary tumor	Upfront surgical resection prior to systemic therapy	Not applicable	GEM	None	Non-comparable	Excluded: no post-acquisition systemic therapy.
**2**	Primary tumor	Upfront surgical resection prior to adjuvant therapy	Before	GEM	GEM	Exact	Included: exact regimen match.
**3**	Primary tumor	Upfront surgical resection prior to systemic therapy	Not applicable	GEM	FFX	Non-comparable	Excluded: non-comparable regimens.
**4**	Primary tumor	Upfront surgical resection prior to adjuvant therapy	Before	GEM	GnP	Approximate	Included: approximate gemcitabine-based match.
**5**	Primary tumor	Surgical resection after neoadjuvant therapy	Before	GEM	GnP	Approximate	Included: approximate gemcitabine-based match.
**5**	Primary tumor	Surgical resection after neoadjuvant therapy	Before	GEM	GnP	Approximate	Included: separate evaluable treatment line.
**6**	Primary tumor	Upfront surgical resection prior to adjuvant therapy	Before	GEM	GEM	Exact	Included: exact regimen match.
**7**	Primary tumor	Upfront surgical resection prior to adjuvant therapy	Before	FFX	FFX	Exact	Included: exact regimen match.
**8**	Primary tumor	Upfront surgical resection prior to adjuvant therapy	Before	FFX	FFX	Exact	Included: exact regimen match.
**9**	Primary tumor	Upfront surgical resection prior to adjuvant therapy	Not applicable	GnP / FFX	GEM	Non-comparable	Excluded: non-comparable regimens.
**10**	Primary tumor	Upfront surgical resection prior to adjuvant therapy	Before	FFX	FFX	Exact	Included: exact regimen match.
**12**	Liver metastasis	Metastatic biopsy prior to systemic metastatic therapy	Before	FFX	FFX	Exact	Included: pretreatment metastatic tissue with exact match.
**13**	Liver metastasis	Metastatic resection after prior chemotherapy exposure	After	FFX	FFX	Exact, post-treatment tissue	Included exploratorily: post-treatment tissue.
**13**	Liver metastasis	Metastatic resection after prior chemotherapy exposure	After	GnP	GnP	Exact, post-treatment tissue	Included: separate exploratory treatment line.
**14**	Liver metastasis	Metastatic biopsy prior to planned systemic therapy	Not applicable	FFX / GnP	None	Non-comparable	Excluded: no post-acquisition systemic therapy.
**17**	Primary tumor	Upfront surgical resection prior to adjuvant therapy	Before	FFX	FFX	Exact	Included: exact regimen match.
**18**	Liver metastasis	Metastatic biopsy prior to planned systemic therapy	Not applicable	FFX / GnP	None	Non-comparable	Excluded: no post-acquisition systemic therapy.

Table S3. Patient-level and treatment-line-level overview of OTSC pharmacotyping cases. Overview of all pharmacotyped cases (n = 15) and cases included in the exploratory clinical correlation cohort (n = 10).

Table S4

Antibody	Clone	Dilution	Catalog number	Manufacturer
Cytokeratin 7	OV-TL 12/30	1:200	M7018	Dako, Düsseldorf, Germany
Ki67	MIB-1	1:200	M7240	Dako, Düsseldorf, Germany
Cleaved caspase 3	5AE1	1:400	9664	Cell Signaling Technology, Inc., Danvers, Massachusetts, USA
Pan-cytokeratin	AE1/AE3	1:200	MS-343	Epredia, Portsmouth NH, USA
α-SMA	1A4	1:400	MS-113	Thermo Scientific, Waltham, Massachusetts, USA
CD3	LN10	1:100	NCL-CD3-565	Novocastra Leica Biosystems, Newcastle upon Tyne, United Kingdom
CD4	4B12	1:25	NCL-L-CD4-368	Novocastra Leica Biosystems, Newcastle upon Tyne, United Kingdom
CD8	C8/144B	1:100	M710301	Dako Denmark A/S, Glostrup, Denmark
CD68	514H12	1:100	NCL-L-CD68	Novocastra Leica Biosystems, Newcastle upon Tyne, United Kingdom
CD163	10D6	1:100	NCL-L-CD163	Novocastra Leica Biosystems, Newcastle upon Tyne, United Kingdom
GATA6	D61E4	1:400	5851S	Cell Signaling Technology, Inc., Danvers, Massachusetts, USA

Table S4: Primary antibodies, dilution and manufacturer information.

Protocol S1. Standard operating procedure for machine-learning-supported digital image analysis of PDAC organotypic tissue slice cultures in QuPath

For detailed assessment of cultivation effects and drug response in OTSCs, sections were scanned as virtual whole-slide-images (WSIs) (3DHistech Panoramic Desk 1.14 slide scanner, 3DHistech, Budapest, Hungary). We assessed morphological parameters semi-automatically using the open access DIA software QuPath (Figure 2C).

Following the preprocessing steps (1; SOP Step 1.4-1.7) estimating stain vectors, automatic tissue detection based on hematoxylin background stain and manual removal of artefacts, the built-in (positive) cell detection command was used to quantify cells (2; SOP Step 1.8). For immunohistochemically stained slides, DAB intensity of cells was quantified. Additional texture and smoothening features for morphological cell cluster/area characterization were calculated (3; SOP Step 1.9).

We established the DIA workflow using a predefined, (i) training (n=64 WSIs), (ii) validation (Ki67 n=108 WSIs, CC3 n=103 WSIs) and (iii) test (n=137 WSIs for Ki67, CC3 respectively) set. Distinct tissue components, i.e., tumor, stroma, necrosis, apoptosis, fibrosis, immune cells, were manually annotated to train staining-specific random-forest classifiers (4; SOP Step 1.10& 1.11). Overall, classifier training comprised 1,196 manually annotated regions across 64 WSIs (Ki67: 344 annotations across 21 WSIs; CC3: 439 annotations across 22 WSIs; H&E: 413 annotations across 21 WSIs). Tumor annotations used for classifier training were guided by virtual alignment of consecutive CK7-stained sections using the interactive image alignment plugin and were additionally verified by visual inspection.

The annotated tissue was divided into 2500 µm2 areas (25*100*100 µm squares) and evaluated as individual data points to standardize the quantified tissue size area (5; SOP Step 1.13). Only areas with a cell count of more than 100 were included to ensure that the cell-based analysis for Ki67 and CC3 had a sufficient number of cells for a percentage-based assessment. The mean area ratio per WSI was used as individual data point for area-based measurements.

Classifier outputs were visually reviewed using QuPath's classification-specific color masks to identify potential misclassifications. In addition, Ki67 and CC3 percentages in tumor-classified regions were independently evaluated by blinded visual scoring (Supplementary Figure S6B&C).

The workflow steps were integrated into groovy scripts to facilitate high-throughput processing and were applied to approximately 250 WSIs per staining across the establishment feasibility and pharmacotyping cohorts, including sections later excluded during histopathological cohort refinement. For downstream pharmacotyping analyses, the workflow was applied to 137 quantified OTSC sections across multiple stainings and treatment conditions.

In addition, we developed two workflows to evaluate the distribution of immune cells around (within) tumor areas. Cells and areas were classified as tumor/stroma or DAB positive (= CD positive) based on adjacent sections stained for PanCK, CK7 and the distinct immune cell markers. This enabled measuring both the distance from the respective immune cell to the nearest tumor cell (1) and the infiltration of immune cells into the tumor areas ((2), Figure 2B).

Resources

Developer's tutorial: https://qupath.readthedocs.io/en/stable/index.html

Program download: https://qupath.github.io/

Original publication: Bankhead, P. et al. *QuPath: Open source software for digital pathology image analysis.* Scientific Reports (2017). https://doi.org/10.1038/s41598-017-17204-5

Overview:

- Step 1.2-1.4: preprocessing
- Step 1.5-1.7: tissue detection
- Step 1.8: cell detection
- Step 1.9-1.11: cell classification
- Step 1.12-1.13: data export & batch processing

1.1 After installing QuPath, create a new Project

1.2 Add new exemplary Images serving as classification input

- Choose “H&E or H-DAB” as the slide was stained with H&E or using DAB-Substrate
- Draw a rectangle somewhere in the image using the rectangle tool
- Click preprocessing→estimate stain vectors

1.3 Estimate Stain Vectors for HE, CK7, Ki67 and CC3 Staining:

setColorDeconvolutionStains('{"Name" : "HE estimated 17780(7) HE", "Stain 1" : "Hematoxylin", "Values 1" : "0.80335 0.55426 0.21779", "Stain 2" : "DAB", "Values 2" : "0.2693 0.50299 0.82127", "Background" : " 240 230 225"}');

setColorDeconvolutionStains('{"Name" : "CK7 H-DAB estimated 17245(6)CK7", "Stain 1" : "Hematoxylin", "Values 1" : "0.817 0.53615 0.21228", "Stain 2" : "DAB", "Values 2" : "0.1344 0.48288 0.86531", "Background" : " 217 213 209"}');

setColorDeconvolutionStains('{"Name" : "H-DAB estimated 17780(15)Ki67", "Stain 1" : "Hematoxylin", "Values 1" : "0.81684 0.55274 0.16505", "Stain 2" : "DAB", "Values 2" : "0.24806 0.57664 0.77843", "Background" : " 243 242 236"}');

setColorDeconvolutionStains('{"Name" : "CC3 H-DAB estimatedm17245(6) CL.Casp3", "Stain 1" : "Hematoxylin", "Values 1" : "0.74482 0.63148 0.21557", "Stain 2" : "DAB", "Values 2" : "0.19476 0.55408 0.80936", "Background" : " 231 225 214"}');

1.4 Create Threshold for Tissue detection:

- Click “Annotations”→ “Add/Remove”→”add class”→type in “tissue”
- Click “Classify”→”pixel classification”→”create thresholder”
- Choose settings as shown in Figure S7, then click ‘create objects’.Double click, then right click on annotated tissue→”annotations”→”unlock”

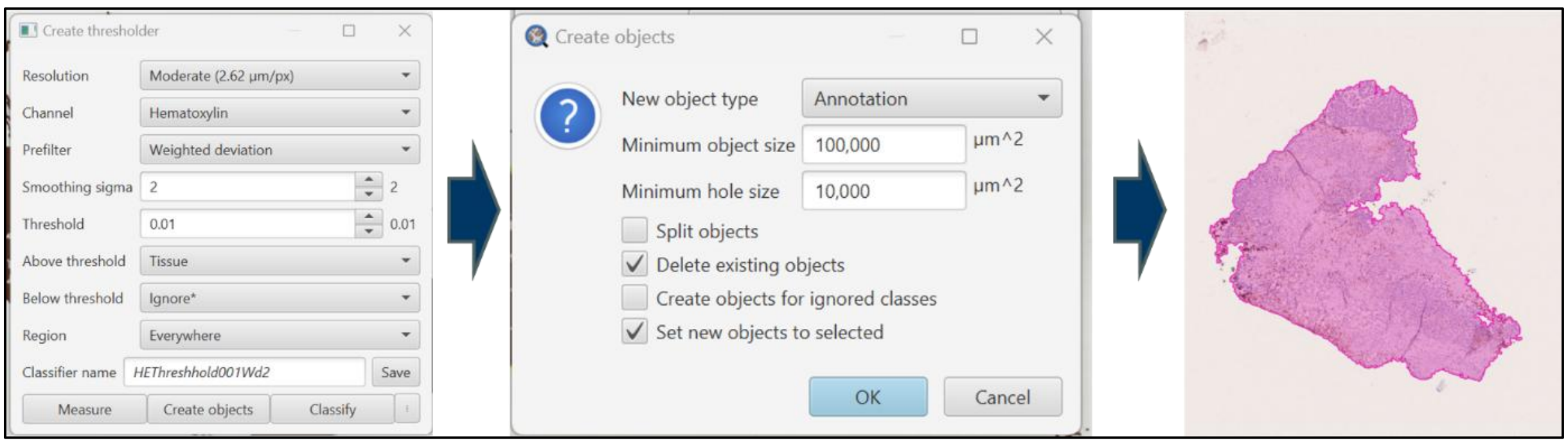

Figure S7. Representative QuPath tissue detection settings used in Protocol S1.

Script:

```
resetSelection();

createAnnotationsFromPixelClassifier("HEThreshold001Wd2", 100000.0, 10000.0, "DELETE_EXISTING", "SELECT_NEW")
```

Name of thresholder for tissue detection:

HEThreshold001Wd2.json

1.5 Run Threshold Detection for Tissue detection and correct manually:

- Manual Correction of Artefacts, e.g. Airbubbles
- Use **brush and wand tool** to annotate and delete annotations (artefacts!) pressing the **alt key simutanously**

1.6 Unlock all Annotations:

```
getAnnotationObjects().each {it.setLocked(false)} fireHierarchyUpdate()
```

1.7 Run Cell Detection command:

- Click “Analyze”→”Cell detection”→”positive cell detection”
- Change “Score compartment” to “Cell: DAB OD mean”
- Change threshold to 0.1
- See Supplementary Figures S8 and S9 for representative workflow and settings.
- Click “Analyze”→”calculate features”→”add intensity features”
- Change Region to “square tiles”
- Tick Hematoxylin (color deconvolved) and compute Haralick features (then measurement maps for visual results (optional))

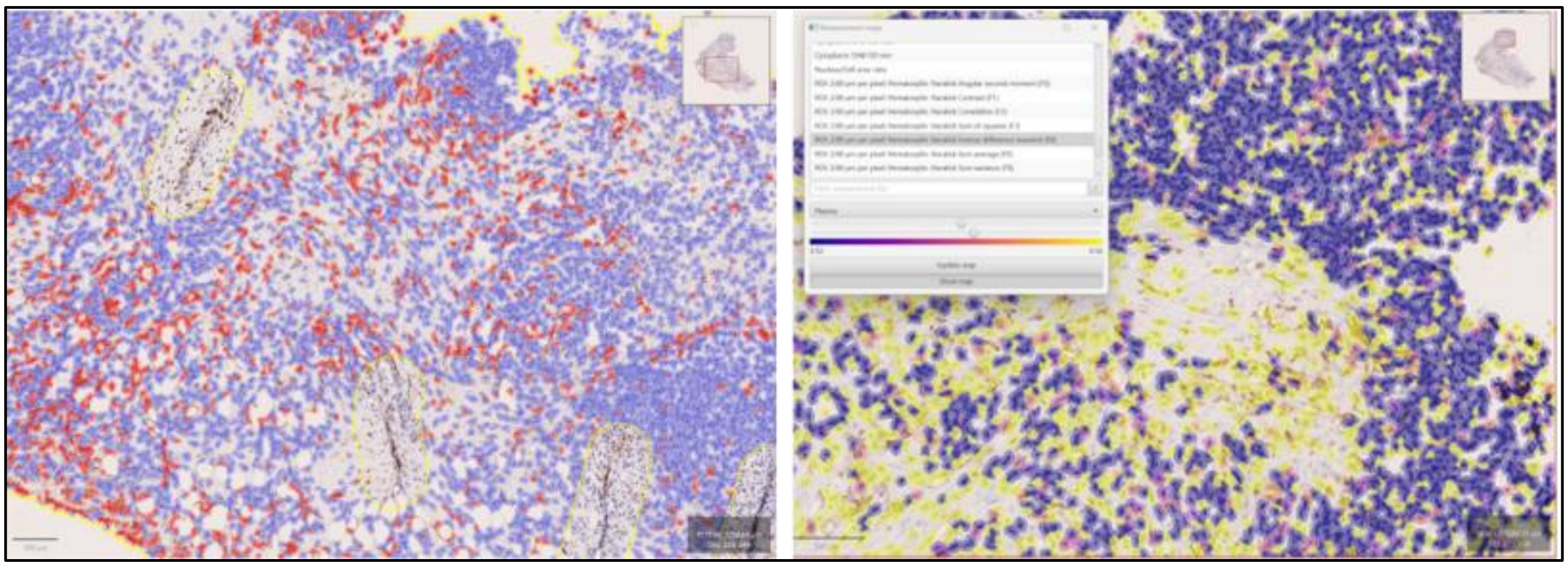

Figure S8. Representative QuPath cell detection and intensity feature calculation used in Protocol S1.

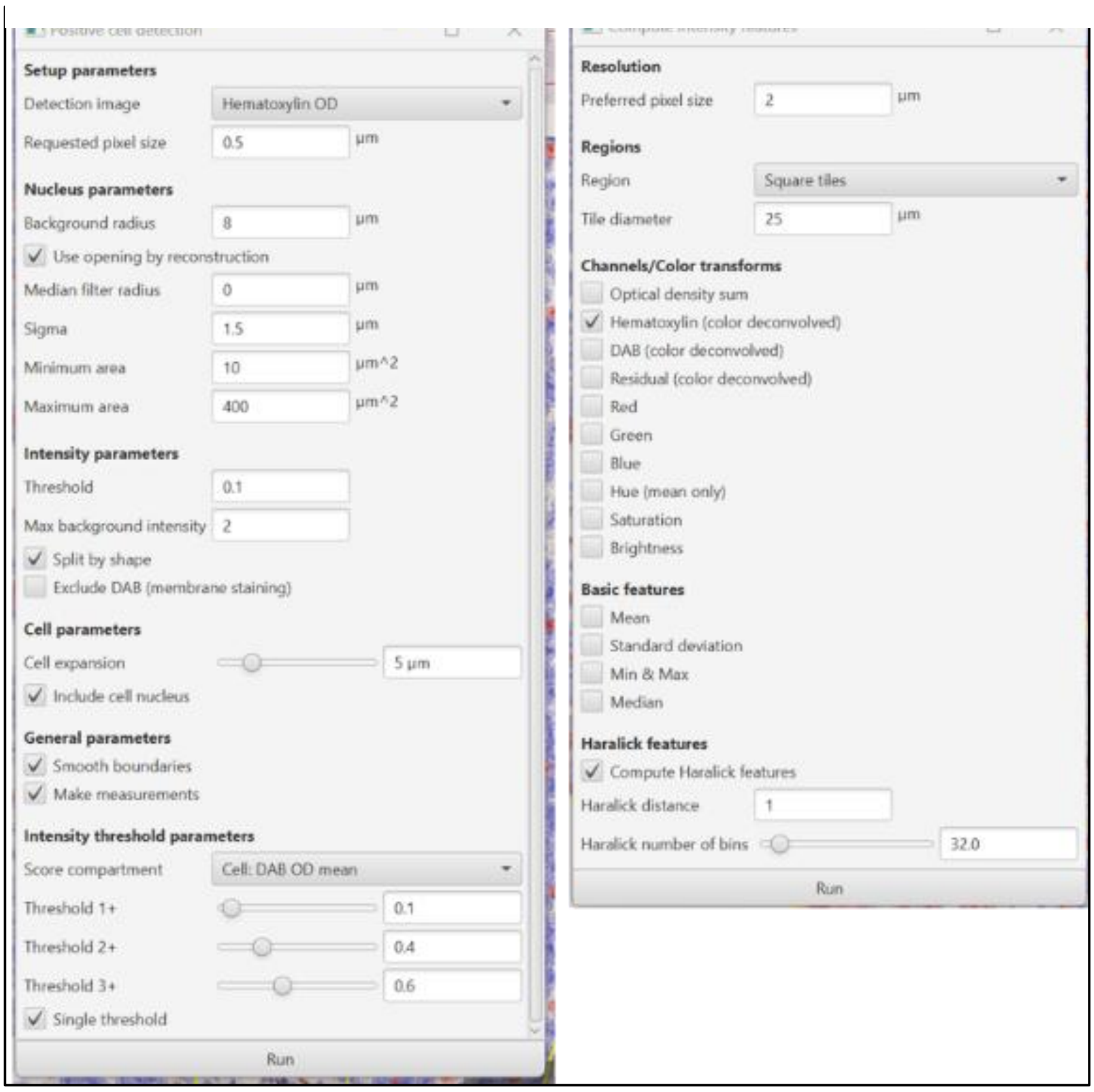

Figure S9. Representative QuPath cell detection and intensity feature settings used in Protocol S1.

Cell Detection Scripts:

HE:

```
selectAnnotations();

runPlugin('qupath.imagej.detect.cells.WatershedCellDetection', '{"detectionImageBrightfield":"Hematoxylin OD","requestedPixelSizeMicrons":0.5,"backgroundRadiusMicrons":8.0,"backgroundByReconstruction":true,"medianRadiusMicrons":0.0,"sigmaMicrons":1.5,"minAreaMicrons":10.0,"maxAreaMicrons":200.0,"threshold":0.13,"maxBackground":2.0,"watershedPostProcess":true,"excludeDAB":false,"cellExpansionMicrons":4.0,"includeNuclei":true,"smoothBoundaries":true,"makeMeasurements":true}')
```

CK7:

```
selectAnnotations();

runPlugin('qupath.imagej.detect.cells.PositiveCellDetection', '{"detectionImageBrightfield":"Hematoxylin OD","requestedPixelSizeMicrons":0.5,"backgroundRadiusMicrons":8.0,"backgroundByReconstruction":true,"medianRadiusMicrons":0.0,"sigmaMicrons":1.5,"minAreaMicrons":10.0,"maxAreaMicrons":200.0,"threshold":0.13,"maxBackground":2.0,"watershedPostProcess":true,"excludeDAB":false,"cellExpansionMicrons":4.0,"includeNuclei":true,"smoothBoundaries":true,"makeMeasurements":true,"thresholdCompartment":"Cytoplasm: DAB OD mean","thresholdPositive1":0.27,"thresholdPositive2":0.4,"thresholdPositive3":0.6000000000000001,"singleThreshold":true}')
```

Ki67:

```
selectAnnotations();

runPlugin('qupath.imagej.detect.cells.PositiveCellDetection', '{"detectionImageBrightfield":"Optical density sum","requestedPixelSizeMicrons":0.5,"backgroundRadiusMicrons":8.0,"backgroundByReconstruction":true,"medianRadiusMicrons":0.0,"sigmaMicrons":1.5,"minAreaMicrons":10.0,"maxAreaMicrons":200.0,"threshold":0.2,"maxBackground":2.0,"watershedPostProcess":true,"excludeDAB":false,"cellExpansionMicrons":4.0,"includeNuclei":true,"smoothBoundaries":true,"makeMeasurements":true,"thresholdCompartment":"Nucleus: DAB OD mean","thresholdPositive1":0.2,"thresholdPositive2":0.3,"thresholdPositive3":0.4,"singleThreshold":false}')
```

CC3:

```
selectAnnotations();

runPlugin('qupath.imagej.detect.cells.PositiveCellDetection', '{"detectionImageBrightfield":"Optical density sum","requestedPixelSizeMicrons":0.5,"backgroundRadiusMicrons":8.0,"backgroundByReconstruction":true,"medianRadiusMicrons":0.0,"sigmaMicrons":1.5,"minAreaMicrons":10.0,"maxAreaMicrons":200.0,"threshold":0.2,"maxBackground":2.0,"watershedPostProcess":true,"excludeDAB":false,"cellExpansionMicrons":4.0,"includeNuclei":true,"smoothBoundaries":true,"makeMeasurements":true,"thresholdCompartment":"Cell: DAB OD mean","thresholdPositive1":0.1,"thresholdPositive2":0.2,"thresholdPositive3":0.3,"singleThreshold":false}')
```

Immune staining:

```
selectAnnotations();
```

```
runPlugin('qupath.imagej.detect.cells.WatershedCellDetection', '{"detectionImageBrightfield":"Hematoxylin OD","requestedPixelSizeMicrons":0.5,"backgroundRadiusMicrons":8.0,"backgroundByReconstruction":true,"medianRadiusMicrons":0.0,"sigmaMicrons":1.5,"minAreaMicrons":10.0,"maxAreaMicrons":200.0,"threshold":0.13,"maxBackground":2.0,"watershedPostProcess":true,"excludeDAB":false,"cellExpansionMicrons":4.0,"includeNuclei":true,"smoothBoundaries":true,"makeMeasurements":true}')
```

1.8 Calculate Intensity features, inclusive Haralick features:

```
selectCells();
```

```
runPlugin('qupath.lib.algorithms.IntensityFeaturesPlugin', '{"pixelSizeMicrons":2.0,"region":"SQUARE","tileSizeMicrons":25.0,"colorOD":true,"colorStain1":true,"colorStain2":true,"colorStain3":true,"colorRed":true,"colorGreen":true,"colorBlue":true,"colorHue":true,"colorSaturation":true,"colorBrightness":true,"doMean":true,"doStdDev":true,"doMinMax":true,"doMedian":true,"doHaralick":true,"haralickDistance":1,"haralickBins":32}')
```

2.5.5. Annotate Tumor/Stroma/Other/Necrosis/Immune classifier using consecutive CK7 Stained Slides of the same samples via alignment plug in:

GitHub - qupath/qupath-extension-align: QuPath extension to interactively align images

Use Interactive Image Alignment to annotate Tumor Areas in Ki67/CC3/HE Schnitten (Plug in, optional)

1.9 Object classification (manual):

- Annotate distinct cell types/tissue types” (two or three ROIs each with brush and wand tool) or
- Click “classify”→”object classification”→”train object classifier”
- Leave default settings and safe it as “classifier”

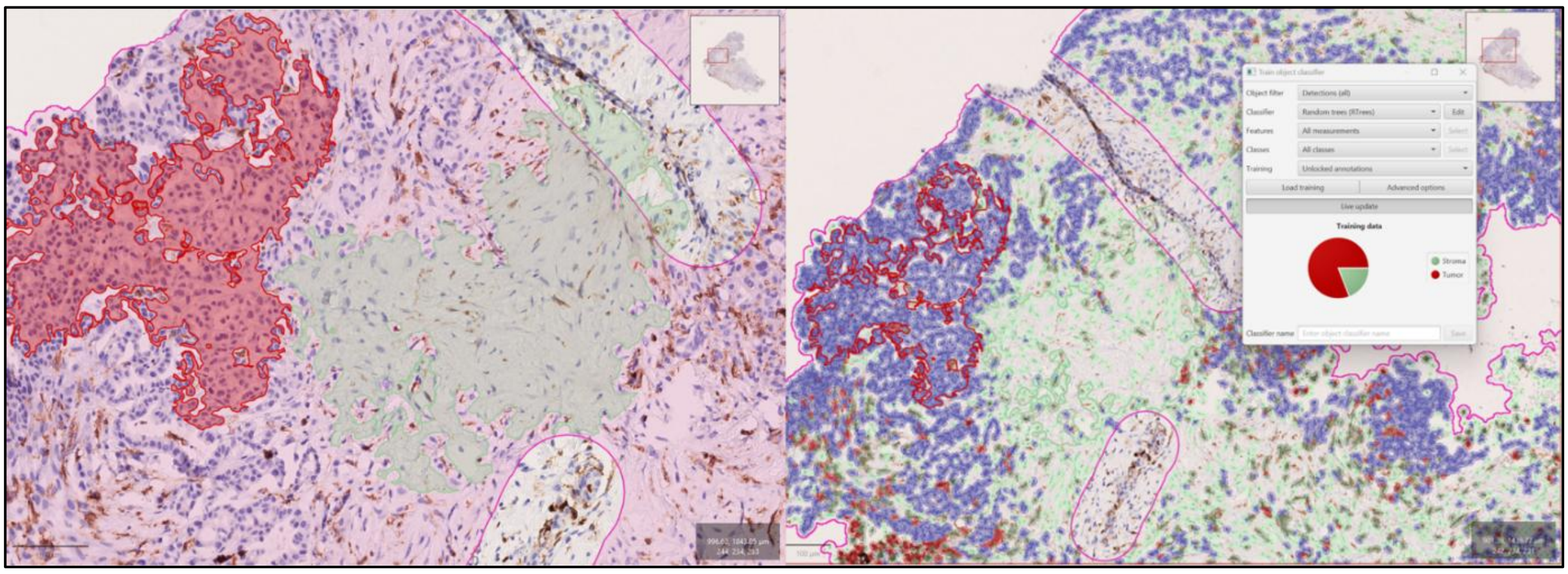

Figure S10. Representative QuPath object-classification training workflow illustrating Protocol S1.

1.10 Build Classifier Tumor/Stroma/(Other)/Immune/Necrosis/Ignore* classifier using the annotations of the different stained slices, run on exemplary slide and save as .json file:

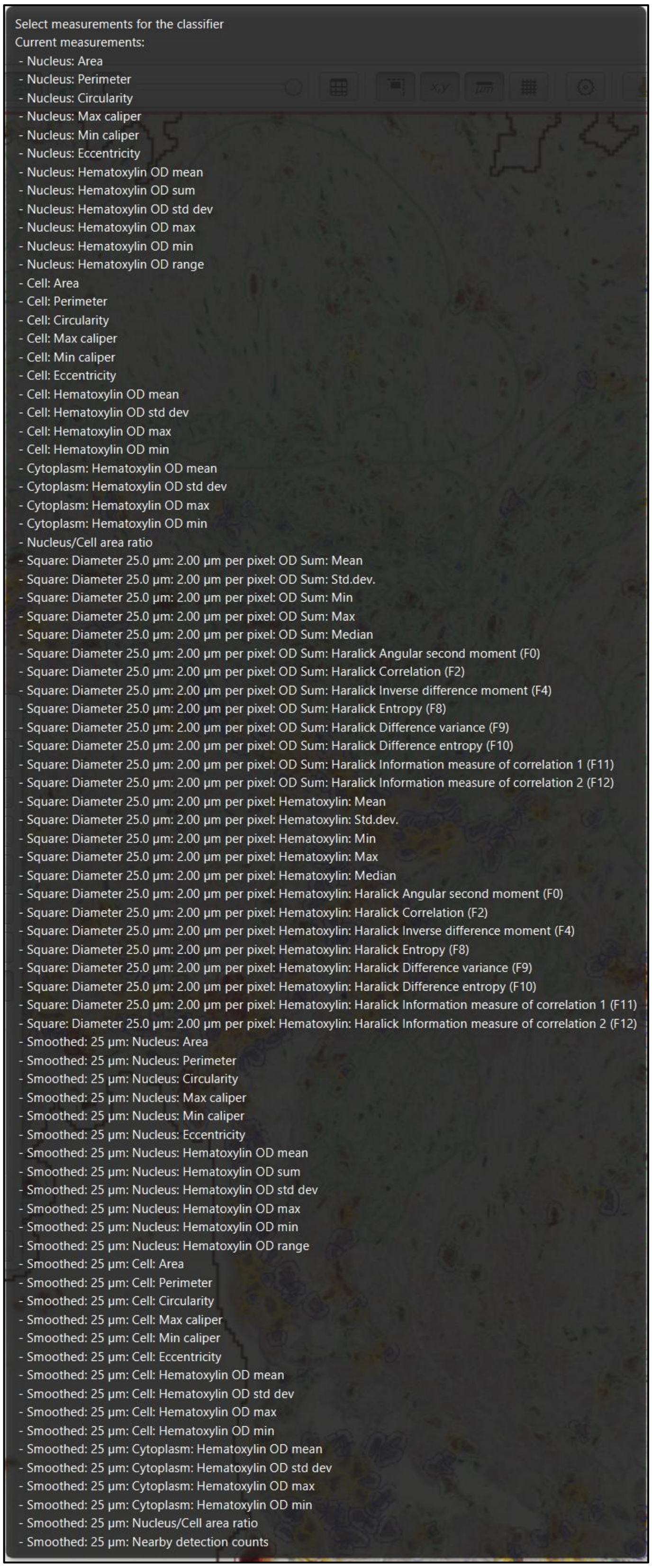

Figure S11. Representative QuPath classifier settings illustrating Protocol S1.

1.11 Data export

- Click “measure”→”export measurements”
- Choose example picture
- Choose “image”
- Choose .csv file
- Export to your project folder.
- Open CSV, save as Excel, and analyze statistically as required.

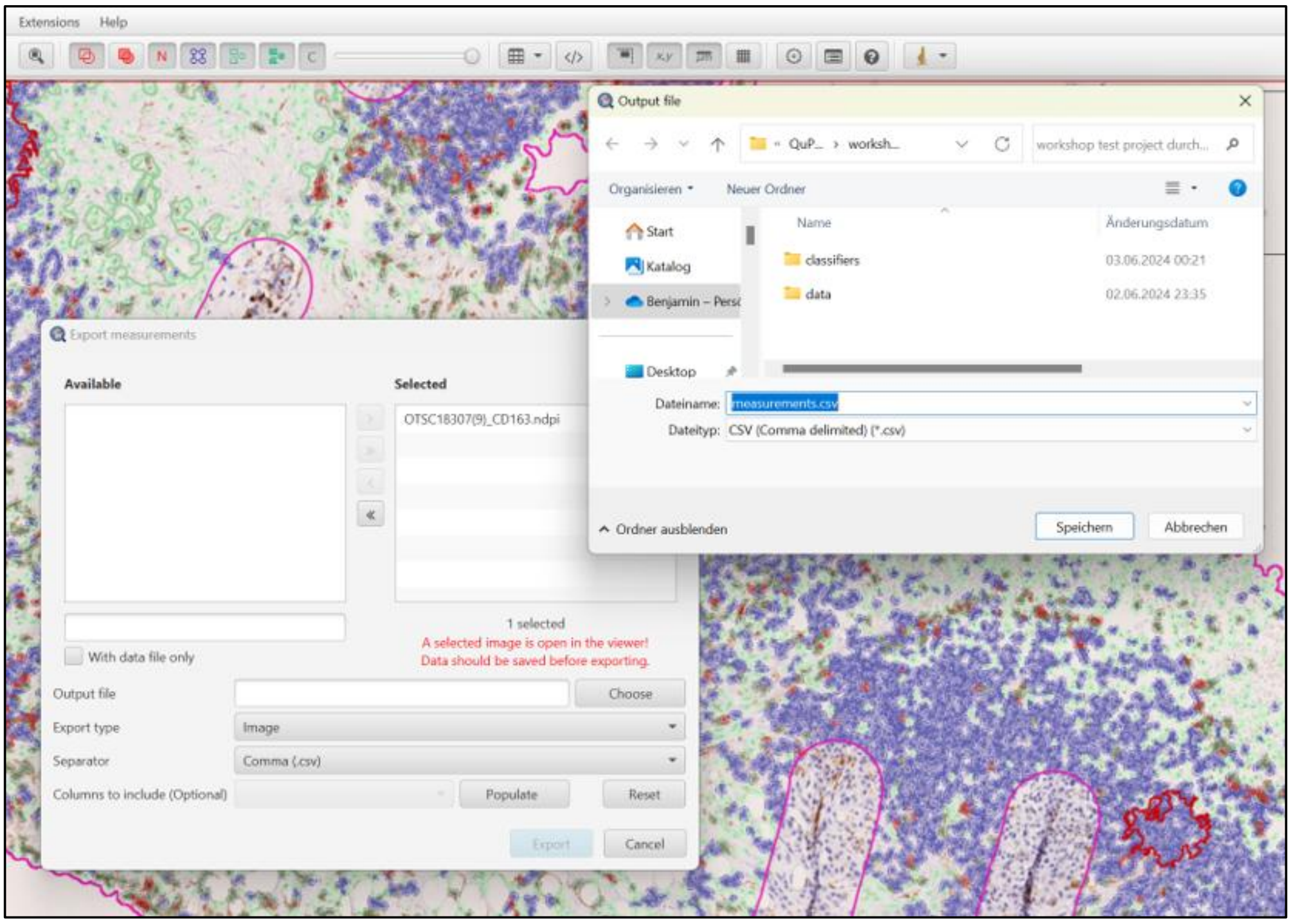

Figure S12. Representative QuPath data export workflow illustrating Protocol S1.

1.12 Batch workflow outline (post-training):

- Estimate stain vectors
- Detect tissue
- Unlock annotations
- Calculate intensity features
- Create annotations from tiles
- Run positive cell detection (Ki67, CC3 or HE)
- Run cell classification (Ki67, CC3 or HE) using the pre-trained classifiers
- Create 100 μm^2 tiles (optional)
- Use 25 tiles equal to 2500 μm^2 area (25*100 μm^2 tiles) and evaluate as individual data points to standardize the quantified tissue size area Include only areas with a cell count of more than 100 to ensure cell-based analysis for Ki67 and CC3 has a sufficient number of cells for a percentage-based assessment. For area-based measurements, use mean area ratio per WSI.
- Export measurements

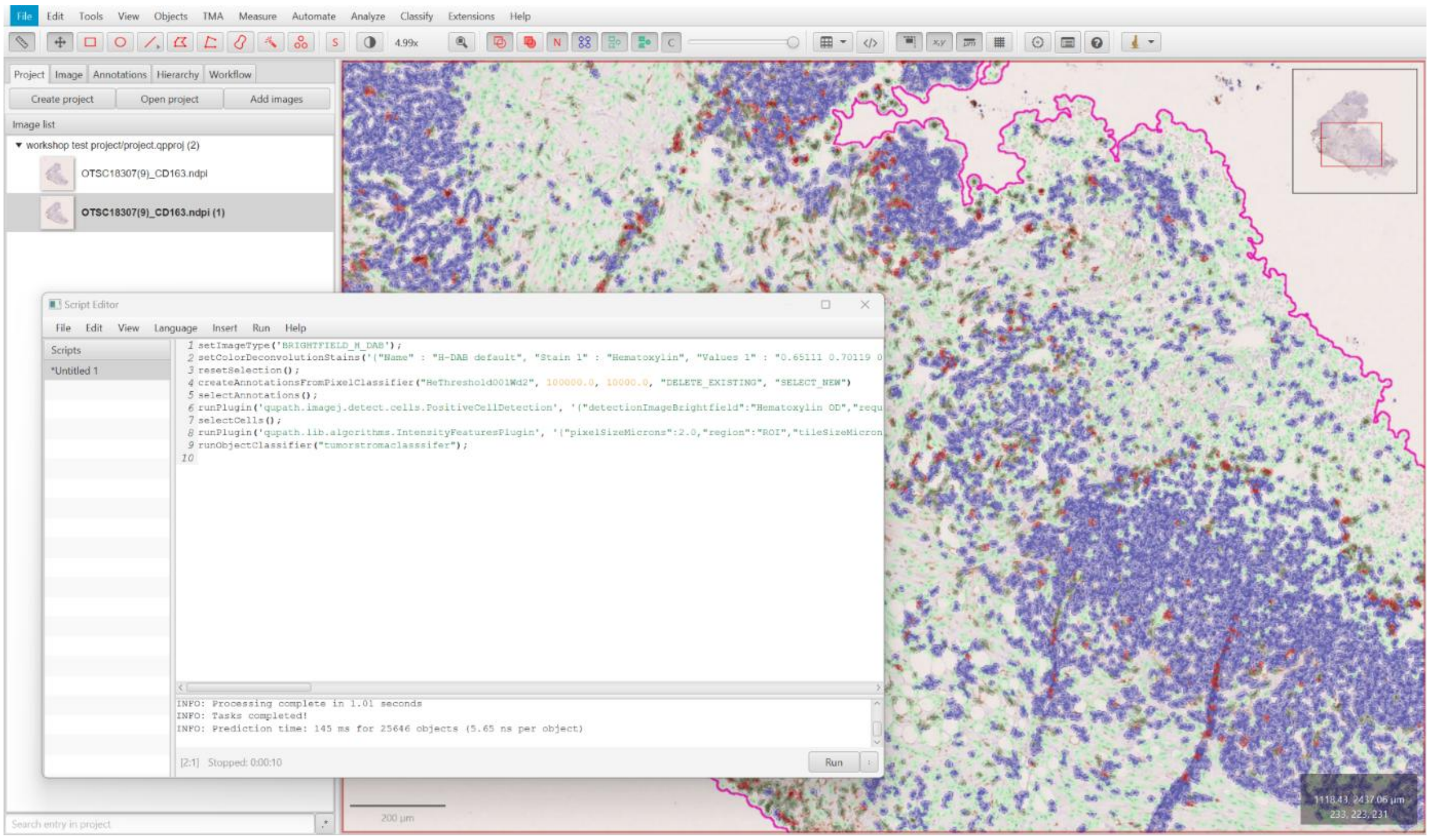

Figure S13. Representative QuPath batch-processing workflow illustrating Protocol S1.

Code S1. QuPath/Groovy scripts for machine-learning-supported digital image analysis of PDAC-OTSCs

Script for CK7 detection (%)

```
setColorDeconvolutionStains('{"Name" : "CK7 H-DAB estimated 17245(6)CK7", "Stain 1" : "Hematoxylin", "Values 1" : "0.817 0.53615 0.21228", "Stain 2" : "DAB", "Values 2" : "0.1344 0.48288 0.86531", "Background" : " 217 213 209"}');

resetSelection();

createAnnotationsFromPixelClassifier("HEThreshold001Wd2", 100000.0, 10000.0, "DELETE_EXISTING", "SELECT_NEW")

runPlugin('qupath.imagej.detect.cells.PositiveCellDetection', '{"detectionImageBrightfield":"Hematoxylin OD","requestedPixelSizeMicrons":0.5,"backgroundRadiusMicrons":8.0,"backgroundByReconstruction":true,"medianRadiusMicrons":0.0,"sigmaMicrons":1.5,"minAreaMicrons":10.0,"maxAreaMicrons":200.0,"threshold":0.13,"maxBackground":2.0,"watershedPostProcess":true,"excludeDAB":false,"cellExpansionMicrons":4.0,"includeNuclei":true,"smoothBoundaries":true,"makeMeasurements":true,"thresholdCompartment":"Cytoplasm: DAB OD mean","thresholdPositive1":0.27,"thresholdPositive2":0.4,"thresholdPositive3":0.6000000000000001,"singleThreshold":true}')

selectAnnotations();

duplicateSelectedAnnotations()

runPlugin('qupath.lib.algorithms.TilerPlugin', '{"tileSizeMicrons":100.0,"trimToROI":false,"makeAnnotations":true,"removeParentAnnotation":false}')

def firstAnnotation = getAnnotationObjects().findAll{it.getPathClass() == getPathClass("Tissue")}

removeObjects(firstAnnotation, true)
```

Script for Ki67 detection in epithelial cells (%)

```
setColorDeconvolutionStains('{"Name" : "H-DAB estimated 17780(15)Ki67", "Stain 1" : "Hematoxylin", "Values 1" : "0.81684 0.55274 0.16505", "Stain 2" : "DAB", "Values 2" : "0.24806 0.57664 0.77843", "Background" : " 243 242 236"}');

resetSelection();

createAnnotationsFromPixelClassifier("HEThreshold001Wd2", 100000.0, 10000.0, "DELETE_EXISTING", "SELECT_NEW")

runPlugin('qupath.imagej.detect.cells.PositiveCellDetection', '{"detectionImageBrightfield":"Optical density sum","requestedPixelSizeMicrons":0.5,"backgroundRadiusMicrons":8.0,"backgroundByReconstruction":true,"medianRadiusMicrons":0.0,"sigmaMicrons":1.5,"minAreaMicrons":10.0,"maxAreaMicrons":200.0,"threshold":0.2,"maxBackground":2.0,"watershedPostProcess":true,"excludeDAB":false,"cellExpansionMicrons":4.0,"includeNuclei":true,"smoothBoundaries":true,"makeMeasurements":true,"thresholdCompartment":"Nucleus: DAB OD mean","thresholdPositive1":0.3,"thresholdPositive2":0.3,"thresholdPositive3":0.4,"singleThreshold":true}')
```

```
selectObjectsByClassification("Tissue")

runPlugin('qupath.lib.plugins.objects.SmoothFeaturesPlugin',
'{"fwhmMicrons":25.0,"smoothWithinClasses":false}')

selectCells();

runPlugin('qupath.lib.algorithms.IntensityFeaturesPlugin',
'{"pixelSizeMicrons":2.0,"region":"SQUARE","tileSizeMicrons":25.0,"colorOD":true,"colorStain
1":true,"colorStain2":true,"colorStain3":true,"colorRed":true,"colorGreen":true,"colorBlue":true
,"colorHue":true,"colorSaturation":true,"colorBrightness":true,"doMean":true,"doStdDev":true,"
doMinMax":true,"doMedian":true,"doHaralick":true,"haralickDistance":1,"haralickBins":32}')

selectCells();

runObjectClassifier("101223 - Ki67 - nointensitybasedmeasurements - no others -
smoothed");

selectAnnotations();

duplicateSelectedAnnotations()

runPlugin('qupath.lib.algorithms.TilerPlugin',
'{"tileSizeMicrons":100.0,"trimToROI":false,"makeAnnotations":true,"removeParentAnnotation
":false}')

def firstAnnotation = getAnnotationObjects().findAll{it.getPathClass() ==
getPathClass("Tissue")}

removeObjects(firstAnnotation, true)
```

Script for CC3 detection in epithelial cells (H-Score)

```
setColorDeconvolutionStains('{"Name" : "CC3 H-DAB estimatedm17245(6) CL.Casp3",
"Stain 1" : "Hematoxylin", "Values 1" : "0.74482 0.63148 0.21557", "Stain 2" : "DAB", "Values
2" : "0.19476 0.55408 0.80936", "Background" : " 231 225 214"}');

resetSelection();

createAnnotationsFromPixelClassifier("HEThreshold001Wd2", 100000.0, 10000.0,
"DELETE_EXISTING", "SELECT_NEW")

runPlugin('qupath.imagej.detect.cells.PositiveCellDetection',
'{"detectionImageBrightfield":"Optical density
sum","requestedPixelSizeMicrons":0.5,"backgroundRadiusMicrons":8.0,"backgroundByReco
nstruction":true,"medianRadiusMicrons":0.0,"sigmaMicrons":1.5,"minAreaMicrons":10.0,"max
AreaMicrons":200.0,"threshold":0.2,"maxBackground":2.0,"watershedPostProcess":true,"excl
udeDAB":false,"cellExpansionMicrons":4.0,"includeNuclei":true,"smoothBoundaries":true,"ma
keMeasurements":true,"thresholdCompartment":"Cell: DAB OD
mean","thresholdPositive1":0.1,"thresholdPositive2":0.2,"thresholdPositive3":0.3,"singleThres
hold":false}')

selectObjectsByClassification("Tissue")

runPlugin('qupath.lib.plugins.objects.SmoothFeaturesPlugin',
'{"fwhmMicrons":25.0,"smoothWithinClasses":false}')

selectCells();
```

```
runPlugin('qupath.lib.algorithms.IntensityFeaturesPlugin',
'{"pixelSizeMicrons":2.0,"region":"SQUARE","tileSizeMicrons":25.0,"colorOD":true,"colorStain1":true,"colorStain2":true,"colorStain3":true,"colorRed":true,"colorGreen":true,"colorBlue":true,"colorHue":true,"colorSaturation":true,"colorBrightness":true,"doMean":true,"doStdDev":true,"doMinMax":true,"doMedian":true,"doHaralick":true,"haralickDistance":1,"haralickBins":32}')

selectAnnotations();

runObjectClassifier("101223 - CC3 - nointensitybasedmeasurements - no others - smoothed");

selectAnnotations();

duplicateSelectedAnnotations()

runPlugin('qupath.lib.algorithms.TilerPlugin',
'{"tileSizeMicrons":100.0,"trimToROI":false,"makeAnnotations":true,"removeParentAnnotation":false}')

def firstAnnotation = getAnnotationObjects().findAll{it.getPathClass() == getPathClass("Tissue")}

removeObjects(firstAnnotation, true)
```

Script for Analysis of spatial distance between immune and epithelial cells (µm) and overall CD-positive cell percentage:

```
setColorDeconvolutionStains('{"Name" : "Immune cells H-DAB estimated 16680-D0_CD8", "Stain 1" : "Hematoxylin", "Values 1" : "0.70983 0.64663 0.27932", "Stain 2" : "DAB", "Values 2" : "0.30624 0.55762 0.77154", "Background" : " 224 220 214"}');

resetSelection();

createAnnotationsFromPixelClassifier("HEThreshold001Wd2", 100000.0, 10000.0, "DELETE_EXISTING", "SELECT_NEW")

selectAnnotations();

runPlugin('qupath.imagej.detect.cells.WatershedCellDetection',
'{"detectionImageBrightfield":"Hematoxylin OD","requestedPixelSizeMicrons":0.5,"backgroundRadiusMicrons":8.0,"backgroundByReconstruction":true,"medianRadiusMicrons":0.0,"sigmaMicrons":1.5,"minAreaMicrons":10.0,"maxAreaMicrons":200.0,"threshold":0.13,"maxBackground":2.0,"watershedPostProcess":true,"excludeDAB":false,"cellExpansionMicrons":4.0,"includeNuclei":true,"smoothBoundaries":true,"makeMeasurements":true}')

selectObjectsByClassification("Tissue")

runPlugin('qupath.lib.plugins.objects.SmoothFeaturesPlugin',
'{"fwhmMicrons":25.0,"smoothWithinClasses":false}')

selectCells();

runPlugin('qupath.lib.algorithms.IntensityFeaturesPlugin',
'{"pixelSizeMicrons":2.0,"region":"SQUARE","tileSizeMicrons":25.0,"colorOD":true,"colorStain1":true,"colorStain2":true,"colorStain3":true,"colorRed":true,"colorGreen":true,"colorBlue":true,"colorHue":true,"colorSaturation":true,"colorBrightness":true,"doMean":true,"doStdDev":true,"doMinMax":true,"doMedian":true,"doHaralick":true,"haralickDistance":1,"haralickBins":32}')
```

```
selectCells();

runObjectClassifier("Classifier_immune_cells_29082024_hepatocytes_ISe");

detectionCentroidDistances(true)
```

Script for CD-positive infiltration in epithelial (tumoral) areas:

```
setColorDeconvolutionStains('{"Name" : "Immune cells H-DAB estimated 16680-D0_CD8", "Stain 1" : "Hematoxylin", "Values 1" : "0.70983 0.64663 0.27932", "Stain 2" : "DAB", "Values 2" : "0.30624 0.55762 0.77154", "Background" : " 224 220 214"}');

resetSelection();

createAnnotationsFromPixelClassifier("HEThreshold001Wd2", 100000.0, 10000.0, "DELETE_EXISTING", "SELECT_NEW")

selectObjectsByClassification("Tissue")

runPlugin('qupath.lib.algorithms.TilerPlugin', '{"tileSizeMicrons":10.0,"trimToROI":true,"makeAnnotations":false,"removeParentAnnotation":false}')

selectDetections();

runPlugin('qupath.lib.algorithms.IntensityFeaturesPlugin', '{"pixelSizeMicrons":2.0,"region":"SQUARE","tileSizeMicrons":25.0,"colorOD":true,"colorStain1":true,"colorStain2":true,"colorStain3":true,"colorRed":true,"colorGreen":true,"colorBlue":true,"colorHue":true,"colorSaturation":true,"colorBrightness":true,"doMean":true,"doStdDev":true,"doMinMax":true,"doMedian":true,"doHaralick":true,"haralickDistance":1,"haralickBins":32}')

selectObjectsByClassification("Tissue")

runPlugin('qupath.lib.plugins.objects.SmoothFeaturesPlugin', '{"fwhmMicrons":25.0,"smoothWithinClasses":false}')

selectObjectsByClassification("Tissue")

runObjectClassifier("TumorStomaTileclassifierforImmunecellpopulations240224");

runPlugin('qupath.lib.plugins.objects.TileClassificationsToAnnotationsPlugin', '{"pathClass":"All classes","deleteTiles":true,"clearAnnotations":true,"splitAnnotations":false}')

selectObjectsByClassification("Tissue")

clearSelectedObjects(true);

selectObjectsByClassification("Tumor")

runPlugin('qupath.imagej.detect.cells.PositiveCellDetection', '{"detectionImageBrightfield":"Hematoxylin OD","requestedPixelSizeMicrons":0.5,"backgroundRadiusMicrons":8.0,"backgroundByReconstruction":true,"medianRadiusMicrons":0.0,"sigmaMicrons":1.5,"minAreaMicrons":10.0,"maxAreaMicrons":200.0,"threshold":0.13,"maxBackground":2.0,"watershedPostProcess":true,"excludeDAB":false,"cellExpansionMicrons":4.0,"includeNuclei":true,"smoothBoundaries":true,"makeMeasurements":true,"thresholdCompartment":"Cell: DAB OD mean","thresholdPositive1":0.13,"thresholdPositive2":0.4,"thresholdPositive3":0.6000000000000001,"singleThreshold":true}')
```

```
selectObjectsByClassification("Stroma")

runPlugin('qupath.imagej.detect.cells.PositiveCellDetection',
'{"detectionImageBrightfield":"Hematoxylin
OD","requestedPixelSizeMicrons":0.5,"backgroundRadiusMicrons":8.0,"backgroundByRecon
struction":true,"medianRadiusMicrons":0.0,"sigmaMicrons":1.5,"minAreaMicrons":10.0,"maxA
reaMicrons":200.0,"threshold":0.13,"maxBackground":2.0,"watershedPostProcess":true,"excl
udeDAB":false,"cellExpansionMicrons":4.0,"includeNuclei":true,"smoothBoundaries":true,"ma
keMeasurements":true,"thresholdCompartment":"Cell: DAB OD
mean","thresholdPositive1":0.13,"thresholdPositive2":0.4,"thresholdPositive3":0.6000000000
000001,"singleThreshold":true}')
```
